# The Influence of Neuronal Density and Maturation on Network Activity of Hippocampal Cell Cultures: A Methodological Study

**DOI:** 10.1371/journal.pone.0083899

**Published:** 2013-12-27

**Authors:** Emilia Biffi, Giulia Regalia, Andrea Menegon, Giancarlo Ferrigno, Alessandra Pedrocchi

**Affiliations:** 1 Neuroengineering and Medical Robotics Laboratory, Department of Electronics, Information and Bioengineering, Politecnico di Milano, Milan, Italy; 2 Advanced Light and Electron Microscopy Bio-Imaging Centre, Experimental Imaging Centre, San Raffaele Scientific Institute, Milan, Italy; Imperial College London, United Kingdom

## Abstract

It is known that cell density influences the maturation process of *in vitro* neuronal networks. Neuronal cultures plated with different cell densities differ in number of synapses per neuron and thus in single neuron synaptic transmission, which results in a density-dependent neuronal network activity. Although many authors provided detailed information about the effects of cell density on neuronal culture activity, a dedicated report of density and age influence on neuronal hippocampal culture activity has not yet been reported. Therefore, this work aims at providing reference data to researchers that set up an experimental study on hippocampal neuronal cultures, helping in planning and decoding the experiments. In this work, we analysed the effects of both neuronal density and culture age on functional attributes of maturing hippocampal cultures. We characterized the electrophysiological activity of neuronal cultures seeded at three different cell densities, recording their spontaneous electrical activity over maturation by means of MicroElectrode Arrays (MEAs). We had gather data from 86 independent hippocampal cultures to achieve solid statistic results, considering the high culture-to-culture variability. Network activity was evaluated in terms of simple spiking, burst and network burst features. We observed that electrical descriptors were characterized by a functional peak during maturation, followed by a stable phase (for sparse and medium density cultures) or by a decrease phase (for high dense neuronal cultures). Moreover, 900 cells/mm^2^ cultures showed characteristics suitable for long lasting experiments (e.g. chronic effect of drug treatments) while 1800 cells/mm^2^ cultures should be preferred for experiments that require intense electrical activity (e.g. to evaluate the effect of inhibitory molecules). Finally, cell cultures at 3600 cells/mm^2^ are more appropriate for experiments in which time saving is relevant (e.g. drug screenings). These results are intended to be a reference for the planning of *in vitro* neurophysiological and neuropharmacological experiments with MEAs.

## Introduction

The culture of dissociated primary central neurons is a common and convenient approach to elucidate the role of several factors on neuronal network features, which can have important fallout on the study of pathological processes mimicked *in vitro*. Indeed, structural and functional features of cultured neuronal networks depend upon several factors including the animal model, the tissue origin, the cell density and the physical and biochemical environment. Moreover, these characteristics evolve over time as imprints of neuronal network differentiation and maturation processes [1].

In this context, the changes of morphological and electrophysiological features of neuronal networks can be easily investigated by means of microscopy, calcium imaging and single cell or multi-site electrophysiological recordings, e.g. patch clamp, MicroElectrodes Arrays (MEAs).

Several works reported the existence of a relationship between the age of the culture and its characteristics, as displayed by connections among neurons or spiking activity [2]–[5]. Regardless of the tissue origin of the culture, at 7 Days *In Vitro* (DIV), cultures generally show a lower synaptic density and less neuronal cell connectivity with respect to older stages, with a peak at 14 DIV [4], which reflects the maturation of the network paralleled by that of the electrophysiological properties. Indeed, at 7 DIV the electrical activity is characterized by only single spikes whereas at 14 DIV networks exhibit an increase in firing rate, a rich and stable burst pattern (i.e. episodes of high frequency spiking) and highly synchronized periods of high frequency activity, encompassing simultaneously different network sites [3], [6].

Furthermore, several works have shown that functional properties of developing neuronal networks are also strongly influenced by cell density. Indeed, cell density affects dendrite morphology and synaptic density, due to variations in cell-to-cell contact, and the global concentration of extrinsic factors [7]–[9]. For example, differences in cortical network maturation, in terms of synapse formation and distribution, due to neuronal network density have been demonstrated [8]. Specifically, it has been proved that, after network maturation, there is an inverse relationship between neuronal density and the synapse-to-neuron ratio. Therefore, neuronal cultures with different cell densities address the network maturation by modulating the number of synapses per neuron and thus the single neuron synaptic transmission. Previtera and colleagues [9] assessed the effects of varying cell densities on dendrite branching patterns, demonstrating that density plays a role in regulating dendrite arborisation in hippocampal cultures. Particularly, neurons showed a decrease in the number of primary and secondary dendrites and in the number of terminal points as the initial plating density was increased. Other works coupled the morphological analysis to electrophysiological attributes as derived by cell-patch recordings and calcium imaging from sparse, medium, and high-density hippocampal cultures [10], [11]. It was demonstrated that plating at different densities affects the connectivity among neurons, such that sparse networks exhibited stronger synaptic connections between pairs of recorded neurons than dense cultures. This was associated to different patterns of spontaneous network activity with enhanced burst size but reduced burst frequency in the sparse cultures [10], [11] and less synchronized activity in the dense cultures [11]. It was described that neuronal density also affect the morphology of the dendrites and spines of these neurons, such that sparse neurons had a simpler dendritic tree and fewer dendritic spines [10]. In addition, Wagenaar and co-workers performed a deep investigation of burst features when changing the neuronal density of cortical cell cultures grown on MEAs [6]. They found that plating density has a profound effect on maturation, which was demonstrated by the highest firing rates and the fastest maturation in the most dense cultures. Moreover, they found that cortical cultures display a very rich and wide repertoire of bursting patterns, suggesting that culture-to-culture variability has to be taken into account.

Although the above mentioned works provide detailed and rich information about the effects of cell density on neuronal activity, a comprehensive and simple report of density and age dependent functional data from hippocampal neuronal cultures is not provided in literature. Accordingly, we decided to follow the effects of both neuronal density and culture age on network electrical functional attributes of maturing hippocampal cultures. We chose to investigate the electrophysiological activity readout by means of MEAs, exploiting the possibility of gathering network phenomena and of performing repeated recordings from the same cultures. In order to avoid biased interpretations due to culture-to-culture variability [6], we collected data from the number of cultures needed for a solid statistic (86) and from different batches, seeded and fed according to standard protocols in our laboratories [5]. We analysed data by means of well-established algorithms [5] to extract a pool of simple electrophysiological descriptors from MEA recordings, which are thoroughly described in the Results section. Finally we tried to derive some indications to support the experimental plans of the researchers. In summary, our aim is to provide a solid benchmark for the comparison of the spontaneous behaviour of hippocampal neuronal networks at a specific cell density in terms of electrophysiological descriptors.

## Materials and Methods

### Substrate Preparation

**Figure 1:**
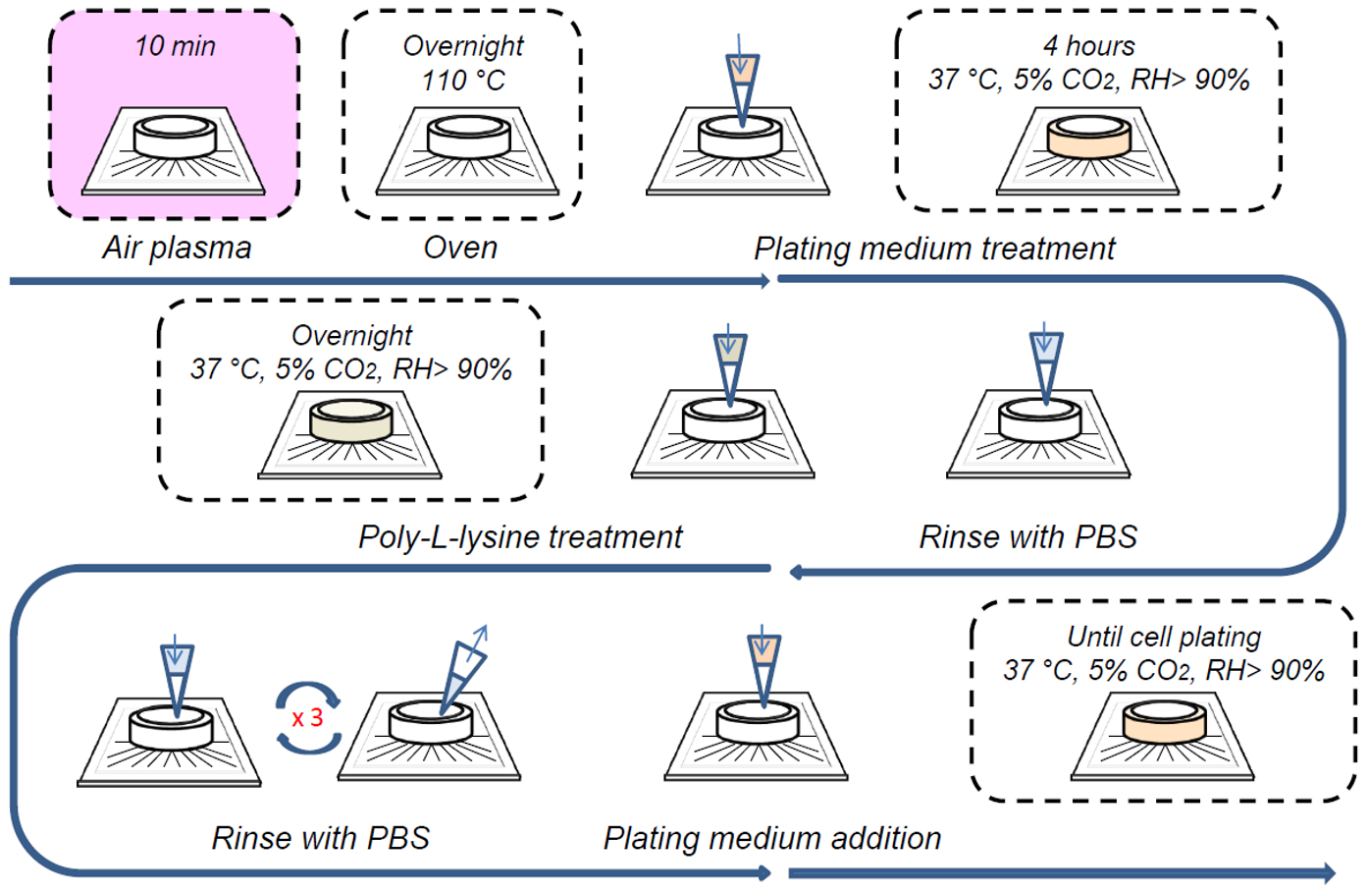
Sequence of the steps to be performed to prepare MEA substrates before cell plating.

We used standard 60-electrode MEA biochips (electrode spacing 200 µm, electrode diameter 30 µm; Multi Channel Systems, MCS GmbH, Germany) as substrate for cell plating. All the reagents and materials used were provided by Invitrogen (Monza, Italy) if not differently otherwise specified. MEA biochips were firstly treated with air plasma for 10 minutes to improve cell adhesion. Then, they were sterilized in an oven at 110°C overnight and treated with plating medium (neurobasal medium (NBM) supplemented with 10% fetal bovine serum (FBS; Lonza, Group Ltd, Basel, Switzerland) and 1% penicillin/streptomycin (P/S)) for 4 h inside a humidified incubator, to increase their hydrophilicity. Afterward, they were treated overnight with poly-L-lysine (2 mg/mL, Sigma–Aldrich srl, Milan, Italy) in 100 mM borate buffer pH 8.5 and placed overnight in a humidified incubator at 37°C. Then, MEAs were carefully rinsed with sterile phosphate buffered saline (PBS) three times to remove the excess of poly-L-lysine and finally incubated overnight with the plating medium, the day before cell plating. The whole procedure is depicted in Figure 1.

### Neuronal Culture Preparation

Mice were maintained in pathogen-free conditions at the San Raffaele Hospital mouse facility (Milan, Italy). All efforts were made to minimize animal suffering and to reduce the number of mice used in accordance with the European Communities Council Directive of November 24, 1986 (86/609/EEC). All animal experimental protocols were approved by the Ethics Review Committee for Animal Experimentation of the Italian Ministry of Health. Procedures were performed according to the guidelines of the Institutional Animal Care and Use Committee of the San Raffaele Scientific Institute (protocol number: 427). Deep anaesthesia was used before animal sacrifice.

Primary neuronal cultures were obtained from CD1 mice at E17.5 [12], [13]. In brief, mice were sacrificed by inhalation of CO_2_, and embryos were removed immediately by caesarean section. Brains were dissected in cold HBSS (Hank’s balanced salt solution; Gibco) supplemented with Glucose 0.6% and 5 mM Hepes pH 7.4 (Sigma–Aldrich). Then, hippocampi of embryos were extracted, rinsed with HBSS twice and treated with Trypsin (0.25%; Sigma-Aldrich; 15 min, 37°C). Afterward, cells were washed three times in HBSS, re-suspended in 5 ml of plating medium (NBM, 10% FBS, 1% P/S) and mechanically dissociated. Dissociated cells were plated at 900, 1800, and 3600 cells/mm^2^ (see Table 1) on pre-treated MEAs and incubated for 4 h at 37°C, each inside a Petri dish filled with water to reduce evaporation. The counting procedure was performed with an haemocytometer, by counting living cells by trypan blue staining. The procedure was repeated twice by two different experimenters. Then, the plating medium was replaced with 1 ml cell culture medium (NBM, B-27 1, 1% P/S, Glutamax 1 mM and 10 mM HEPES pH 7.4 (Lonza)). Neuronal cultures were grown and maintained in a humidified incubator up to 32 DIV. The 30% of the total amount of medium was changed every 2 days, 1 hour before each electrophysiological experiment.

**Table 1:** Plating parameters of the cultures used in this work.

*Plating Parameters*			
Nominal density (cells/mm^2^)	900	1800	3600
Number of cultures	13	48	25
Number of batches	3	5	4
Plating volume (µl)	190	380	760
Number of cells plated (nominal)	250000	500000	1000000
Density of suspension (×10^3^ cells/µl)	1.32	1.32	1.32

### Electrophysiological Recordings

The spontaneous electrical activity of 86 independent cell cultures, plated at three different cell densities, was recorded from day 4 up to day 32 *in vitro*. Extracellular recordings were carried out at 37°C with a setup provided by Multi Channel Systems encompassing a pre-amplifier stage (MEA-1060-Inv-BC-Standard, gain: 55, bandwidth: 0.02 Hz–8.5 kHz, MCS GmbH), an amplification and filtering stage (FA64S, gain 20, bandwidth: 10Hz-3kH, MCS GmbH) and a data acquisition device. Raw data were recorded using a sampling frequency equal to 25kHz. Single recordings started right after the stabilization of the electrical signal, i.e. 10 minutes after moving each MEA from the incubator to the recording setup, minimizing the interference of mechanical disturbances [6]. Each recording lasted 10 minutes and was repeated every two days for each neuronal culture. MEA data are available upon request. Please contact alessandra.pedrocchi@polimi.it.

### Data Analysis

After raw data recording, the spike detection was performed using a different threshold for each channel, which was set equal to a multiple of the standard deviation of average noise amplitude computed during 500 ms at the beginning of each measurement. The chosen threshold is the standard used in our laboratory during MEA experiments with hippocampal dissociated cultures [14], [15], i.e. five times the basal noise. This threshold ensures the detection of few false positive and negative events during the spike detection procedure. No spike sorting procedure was carried out. The following off-line analyses, performed on multiunit data, were implemented in Matlab (The Mathworks, Natick, MA, USA) and many descriptors of the electrophysiological activity were extracted by means of standard algorithms [3], [5], [14], [15]. The selected parameters were identified from a strong literature on this topic [3], [14], [15]. Then, we extracted features describing the simple spiking, burst and network burst (NB) activity. Bursts, defined as episodes of high frequency spiking, were detected when the minimum number of spikes was equal to 10 and the maximum inter spike interval was 100 ms [14], [15]. Network bursts, which are recurrent events of synchronized firing occurring in different electrodes, were identified when the product of the number of active channels and the number of spikes was bigger than 9 and when the minimum inter network burst interval was 100 ms, as previously described [3], [14], [15]. Specifically, to characterize the spiking activity, we extracted the number of channels with activity higher than 0.03 Hz, normalized over the total number of channels (i.e. number of active channels), and the mean frequency of the spiking activity of each neuronal network. In order to describe the burst activity, we extracted the number of channels showing bursts, the burst duration, the intra burst frequency (i.e. the mean frequency inside bursts), and the bursting rate. Finally, to measure the NB activity, we extracted the length of NB, the mean frequency inside the NB (intra NB frequency), the rate of occurrence of NBs (i.e. network bursting rate) and the percentage of spikes involved in NBs.

### Statistical Analysis

First we verified the distribution of each parameter by performing a Lilliefors test. Since our data showed a non-Gaussian distribution, we performed a non-parametrical analysis of variance with a one-way Friedman test for repeated measures over the DIV (i.e. grouping all the cultures by DIV and neglecting the cell density values). When the Friedman test was significant, a Wilcoxon matched pair test was performed between groups. Moreover, we performed a non-parametrical analysis of variance with a one-way Kruskal-Wallis test for independent data over the densities (i.e. grouping all the cultures by the cell density value and neglecting the DIV of observation). When the Kruskal-Wallis test was significant, a Mann-Whitney U test was performed between groups. In the Results section, data are expressed as medians and percentiles, e.g. median +Δ^+^ – Δ^−^ (where Δ^+^ = 75° percentile –median and Δ^−^ = |25° percentile-median|). Statistical analyses were performed with the software Statistica (StatSoft, Inc., Tulsa, OK, USA). The significance level was set at p<0.05.

## Results

Hippocampal neurons were cultured up to 5 weeks. Cells started emitting neurites few hours after plating, forming networks within a few days (i.e. 3–7 depending on the cell culture density), as judged by optical observations.

In the following subsections, we report data describing the spontaneous electrical activity of 86 independent cell cultures, measured every two days starting from day 4 up to day 32 *in vitro*. The data set concerning each analysed parameter, is formed by a number of rows equal to the number of observed cultures (i.e. 86) and a number of columns equal to the number of observed DIV (i.e. 15 repeated measures). Moreover, every row is identified by a certain cell density that represents the grouping variable (i.e. three grouping variables).

### Features of Spiking Activity

**Figure 2:**
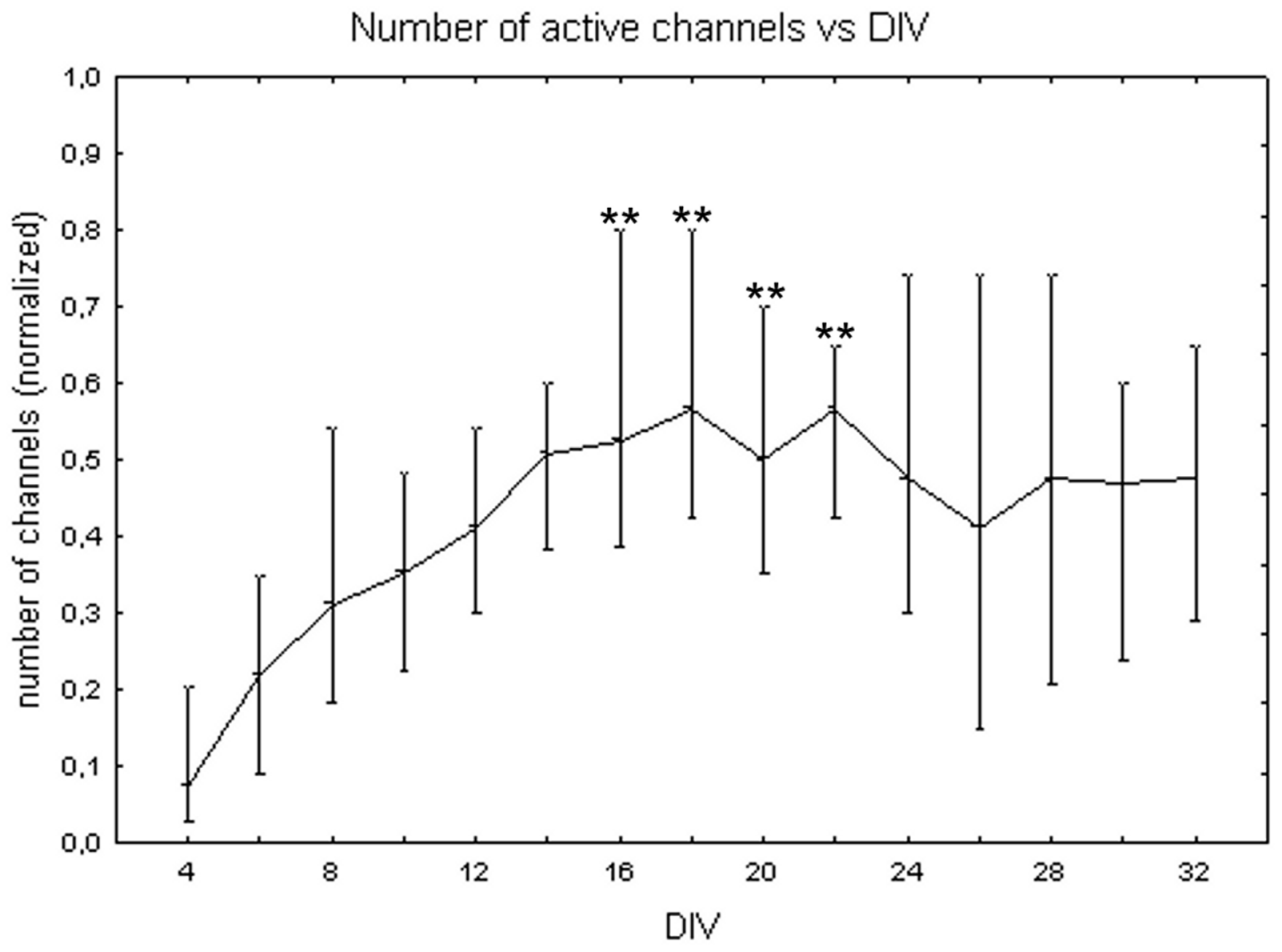
Changes of the number of active channels during cell culture maturation. Median values include different cell densities for each DIV. Values are normalized over the total number of channels. One-way Friedman test and post-hoc Wilcoxon matched pair test **p<0.01 with respect to DIV 4 and 6.

**Figure 3:**
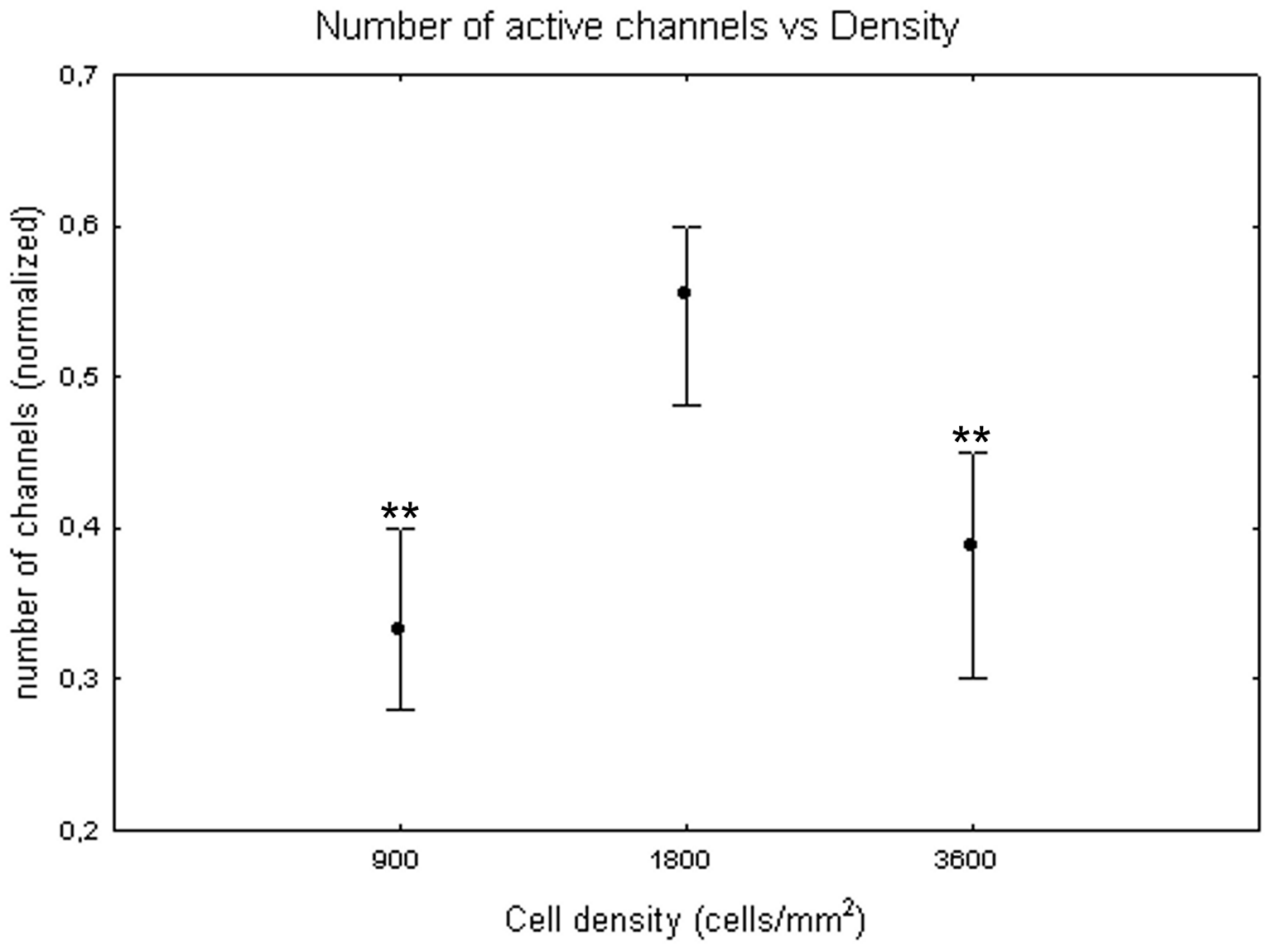
Median number of active channels at 900, 1800 and 3600 cells/mm^2^. Median values include different DIV for each cell density. Values are normalized over the total number of channels. Kruskal-Wallis test and post-hoc Mann-Whitney U test **p<0.01 with respect to cell density of 1800 cells/mm^2^.

**Figure 4:**
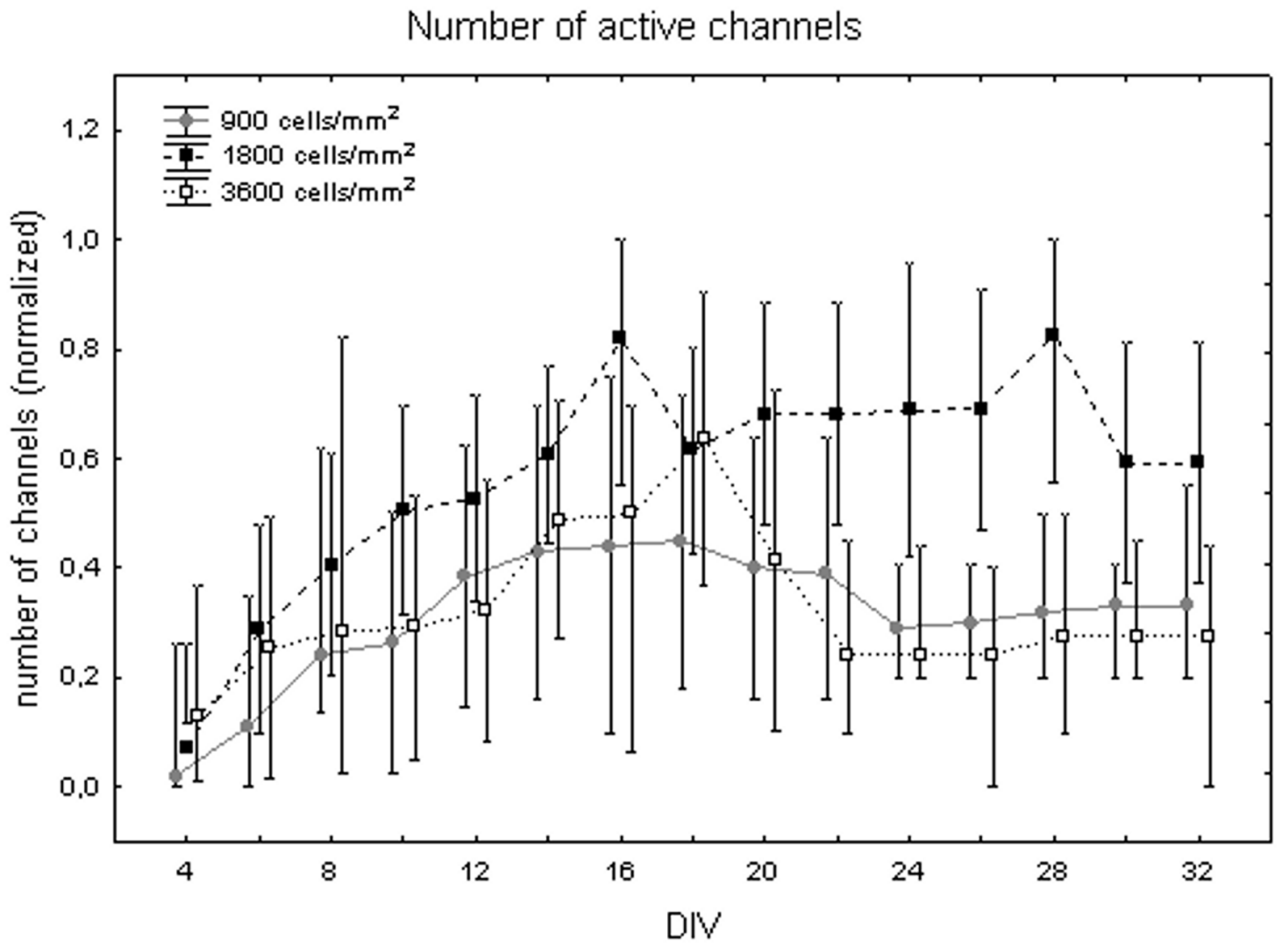
Trend of the number of active channels during cell culture maturation at different cell densities. Cell culture density equal to 900 cells/mm^2^ (grey dots), 1800 cells/mm^2^ (black squares) and 3600 cells/mm^2^ (white squares). Values are normalized over the total number of channels.

Figure 2 shows the number of active channels at different DIV. Values are in the [0, 1] range since the number of active channels was normalized on the total number of channels in each cell culture, as described in the Materials and Methods section. The number of active channels increases over cell maturation reaching its maximum during the third week *in vitro*. The one-way Friedman test for repeated measures showed significant differences between DIV (p<0.01) and the Wilcoxon matched pair test highlighted that these differences are between the first week (DIV 4 and 6) and the third week (DIV 16, 18, 20, 22). The number of active channels also depends on plated cell density. Indeed, the Kruskal-Wallis test identified significant differences among cultures at different cell densities. Specifically, cell cultures at 1800 cells/mm^2^ (0.55+0.07 −0.05) differ from cell cultures at 900 cells/mm^2^ (0.33+0.07 −0.05, Mann-Whitney U test p<0.01) and at 3600 cells/mm^2^ (0.39+0.09 −0.06, Mann-Whitney U test p<0.01) as shown in Figure 3. The quantitative analysis of the trend for each cell density, showed that, in cell cultures with 1800 cells/mm^2^, the number of active channels increased faster, reached higher values than in the other densities and then remained stable (Figure 4, black squares). By contrast, cell cultures with density equal to 3600 cells/mm^2^ showed a reduction of the number of active channels after the end of the second week (Figure 4, white squares). Finally, cell cultures with 900 cells/mm^2^ were characterised by a slower increase in the number of active channels, with a peak around 14 DIV, followed by a stable value (Figure 4, grey dots).

**Figure 5:**
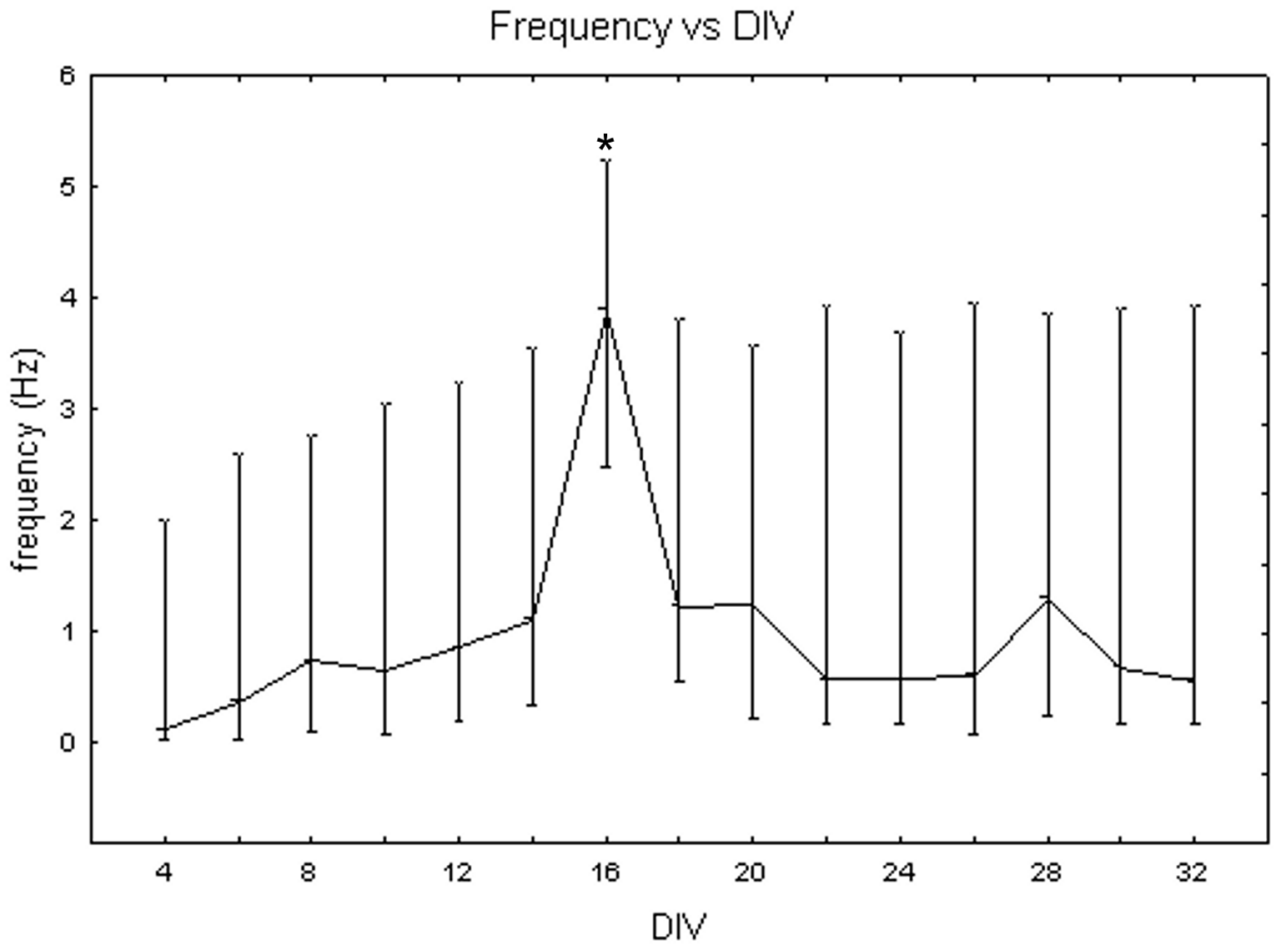
Changes of the mean frequency (Hz) of neuronal networks during cell culture maturation. Median values include different cell densities for each DIV. One-way Friedman test and post-hoc Wilcoxon matched pair test *p<0.05 with respect to all the other days in culture.

**Figure 6:**
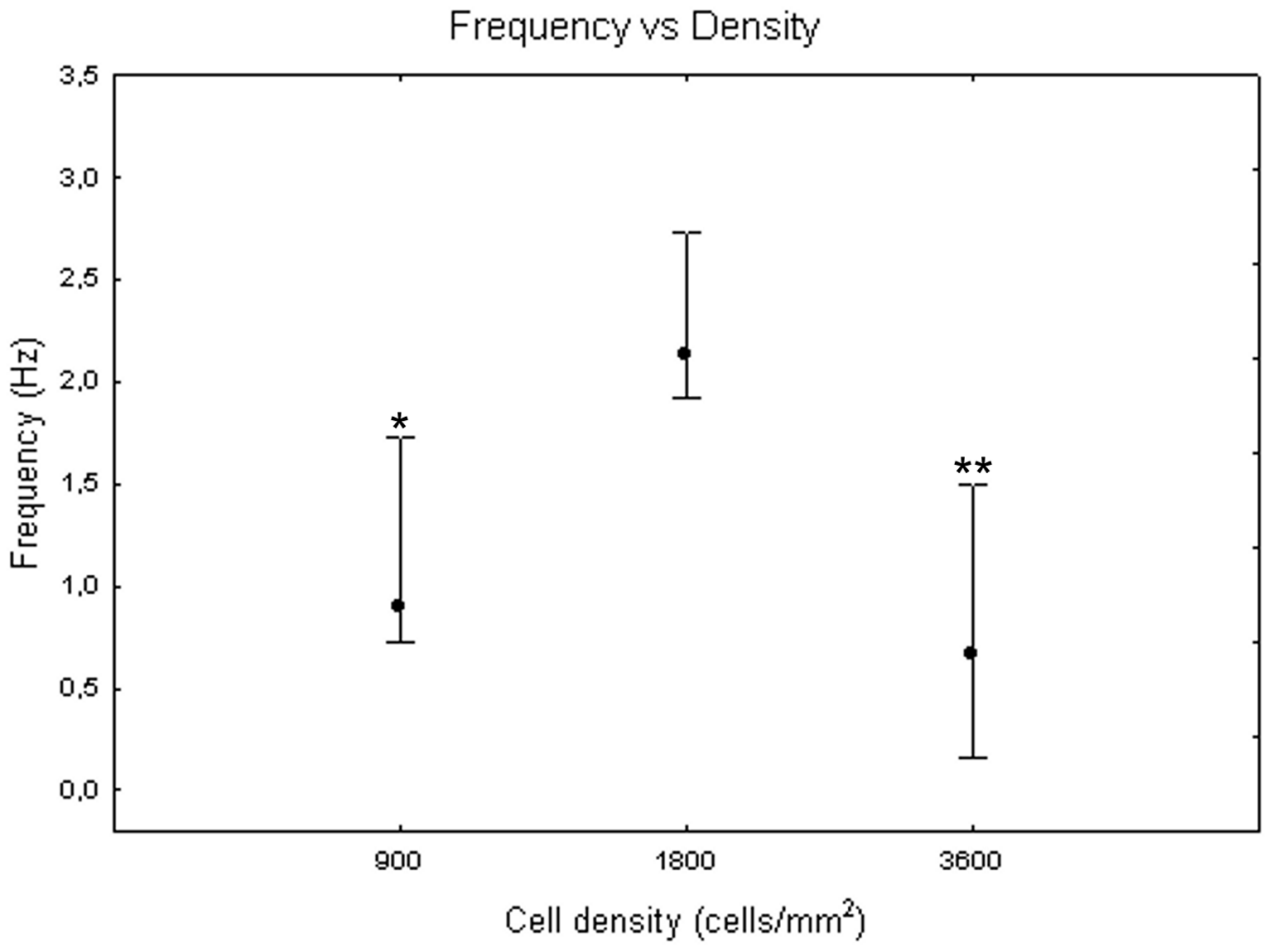
Mean frequency at 900, 1800 and 3600 cells/mm^2^. Median values include different DIV for each cell density. Kruskal-Wallis test and post-hoc Mann-Whitney U test *p<0.05 and **p<0.01 with respect to cell density of 1800 cells/mm^2^.

**Figure 7:**
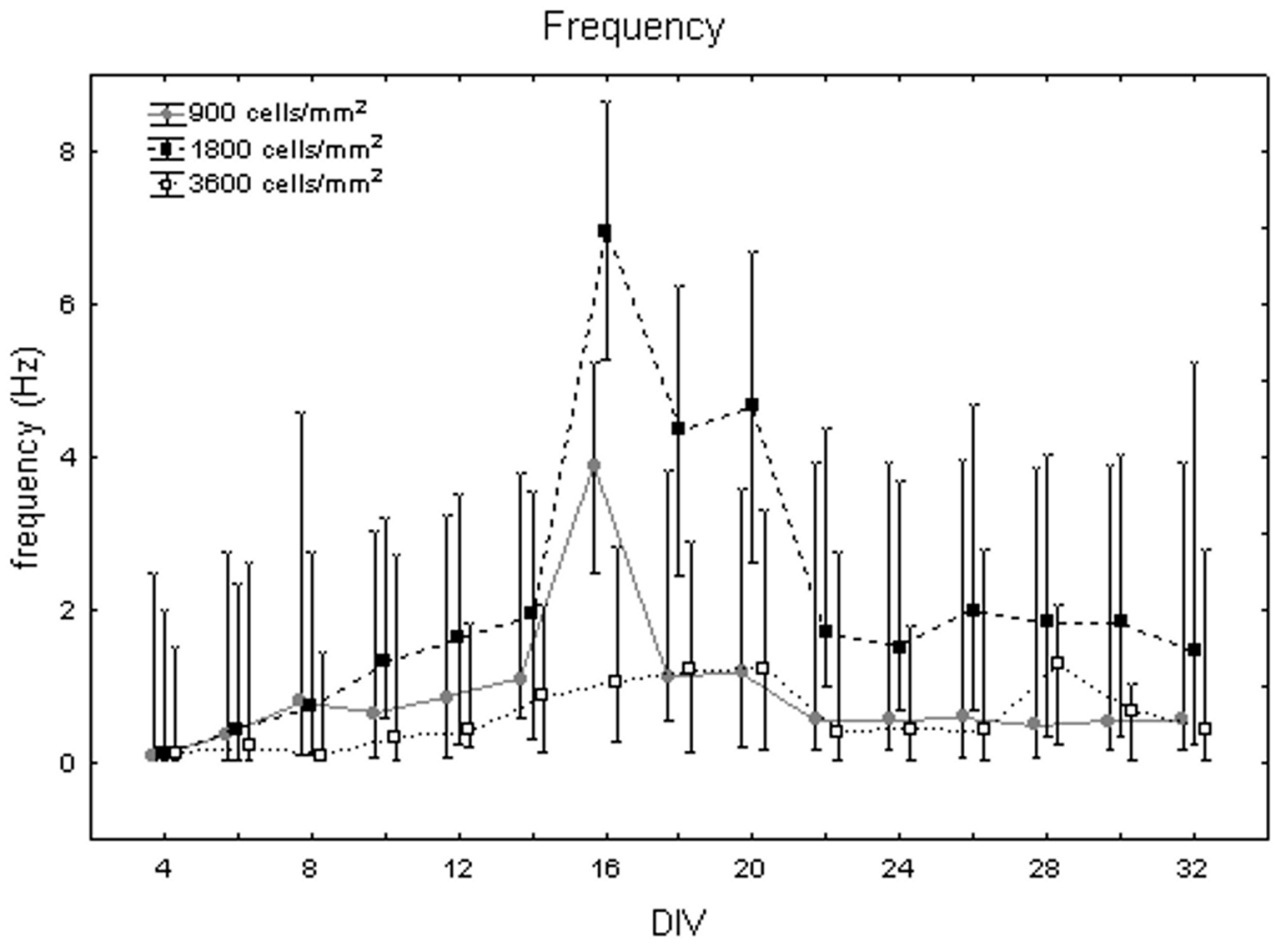
Mean frequency (in Hz) changes during cell culture maturation at different cell densities. Cell culture density equal to 900 cells/mm^2^ (grey dots), 1800 cells/mm^2^ (black squares) and 3600 cells/mm^2^ (white squares).

Figure 5 depicts the mean frequency of neuronal activity at different DIV. It increases during the first and the second weeks, with a peak at 16 DIV. The one-way Friedman for repeated measures showed significant differences between DIV (p<0.05) and the Wilcoxon matched pair test highlighted that these differences are between DIV 16 and all the other days of culture. The variability around median values is high (i.e., more than 3 Hz in many instances), due to the influence of cell density on frequency values. Indeed, the Kruskal-Wallis test identified significant differences between cultures at different cell densities. Specifically, cell cultures at 1800 cells/mm^2^ (2.13+0.61 −0.21 Hz) differ from cell cultures at 900 cells/mm^2^ (0.89+0.84 −0.17 Hz, Mann-Whitney U test p<0.05) and at 3600 cells/mm^2^ (0.67+0.83 −0.52 Hz, Mann-Whitney U test p<0.01), as shown in Figure 6. Analysing qualitatively the trend of frequency values for each cell density, we observed that, during the third week *in vitro*, neuronal cultures at 900 and 1800 cells/mm^2^ reached their highest value of 4 Hz and 7 Hz, respectively (Figure 7, grey dots and black squares, respectively). By contrast, cell cultures at 3600 cells/mm^2^ displayed a smoother trend (Figure 7, white squares). During the fifth week, however, the mean frequency values of cell cultures at 900 and 3600 cells/mm^2^ became similar, while cell cultures at 1800 cells/mm^2^ showed a decrease but still maintained higher values.

### Burst Features

The percentage of channels showing burst activity increases with cell maturation and reaches its maximum value during the third week *in vitro* (Friedman test, p>0.05). It has similar trends for 900 cells/mm^2^ and 1800 cells/mm^2^ cell cultures, remaining stable till the fifth week, while cell cultures at 3600 cells/mm^2^ display a fast decrease in the number of channels showing bursts during the fourth week. Overall, the median value of this descriptor at each density, grouping all the DIV together, is equal to 35% +5% −10%, 44% +3% −14% and 21% +4% −8% for neuronal cultures with cell density of 900 cells/mm^2^, 1800 cells/mm^2^ and 3600 cells/mm^2^, respectively (Kruskal-Wallis test, p>0.05).

**Figure 8:**
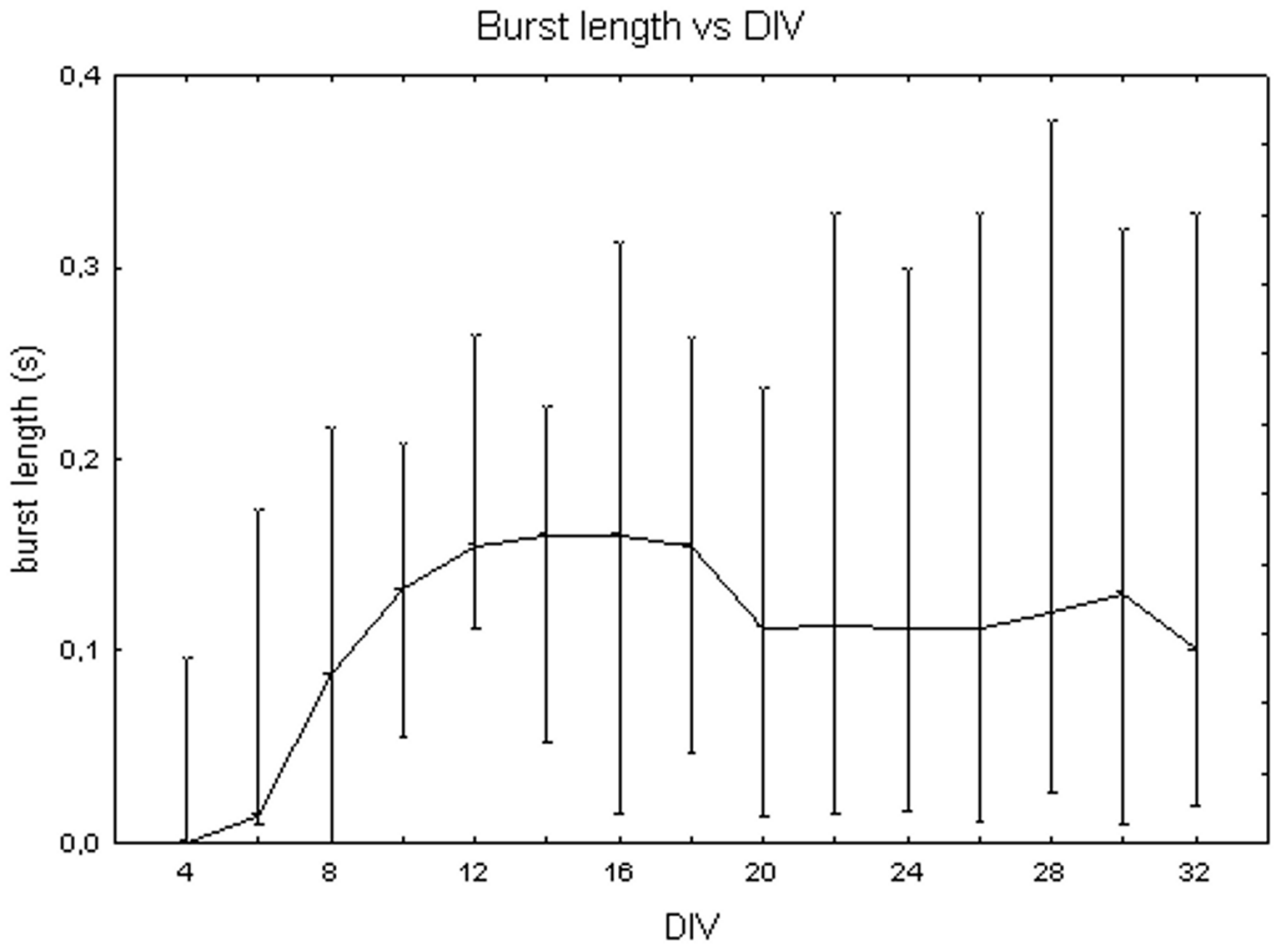
Changes of the burst duration (s) during cell culture maturation. Median values include different cell densities for each DIV.

**Figure 9:**
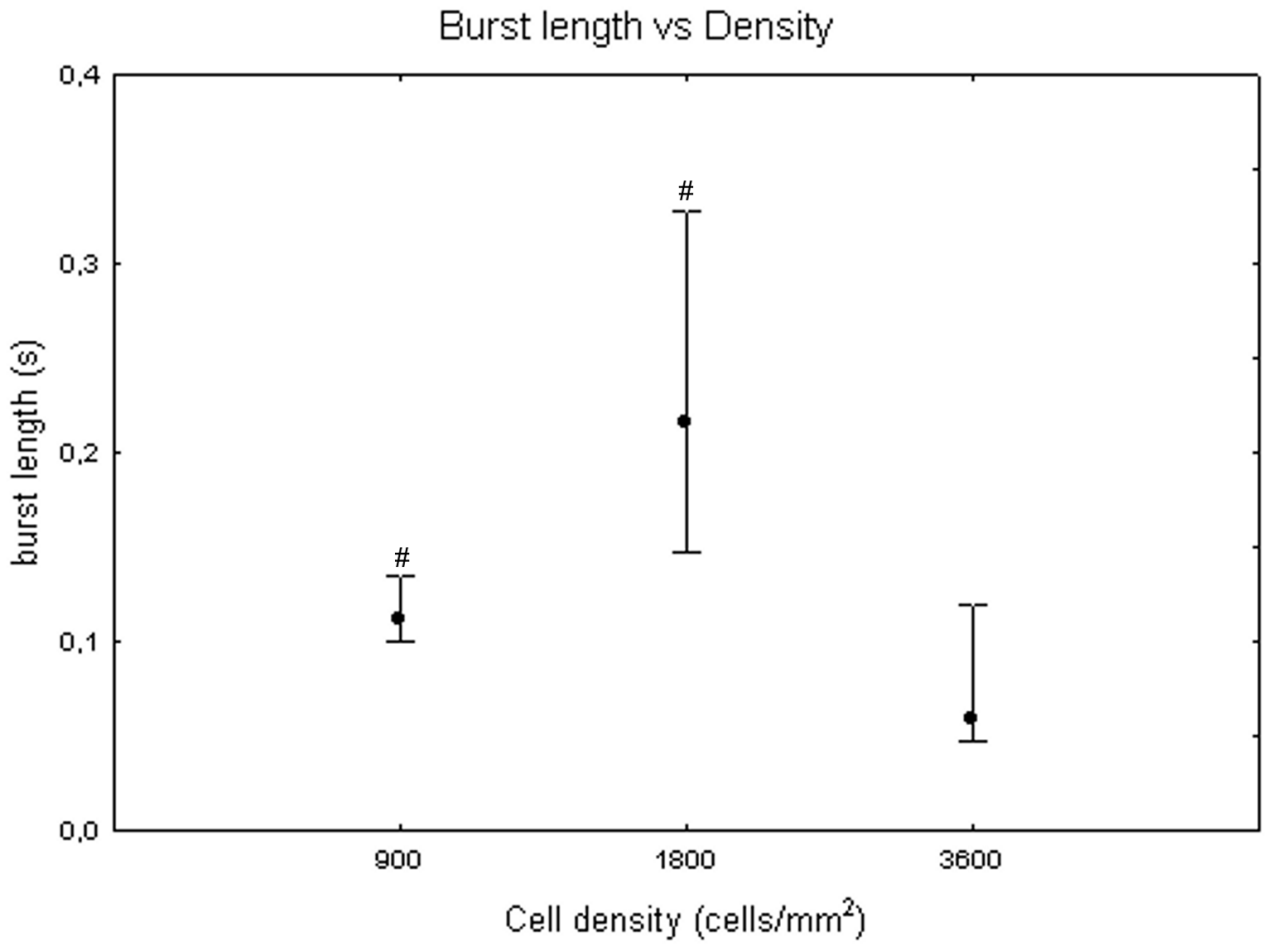
Burst length at 900, 1800 and 3600 cells/mm^2^. Median values include different DIV for each cell density. Kruskal-Wallis test and post-hoc Mann-Whitney U test #p<0.05 with respect to cell density of 3600 cells/mm^2^.

**Figure 10:**
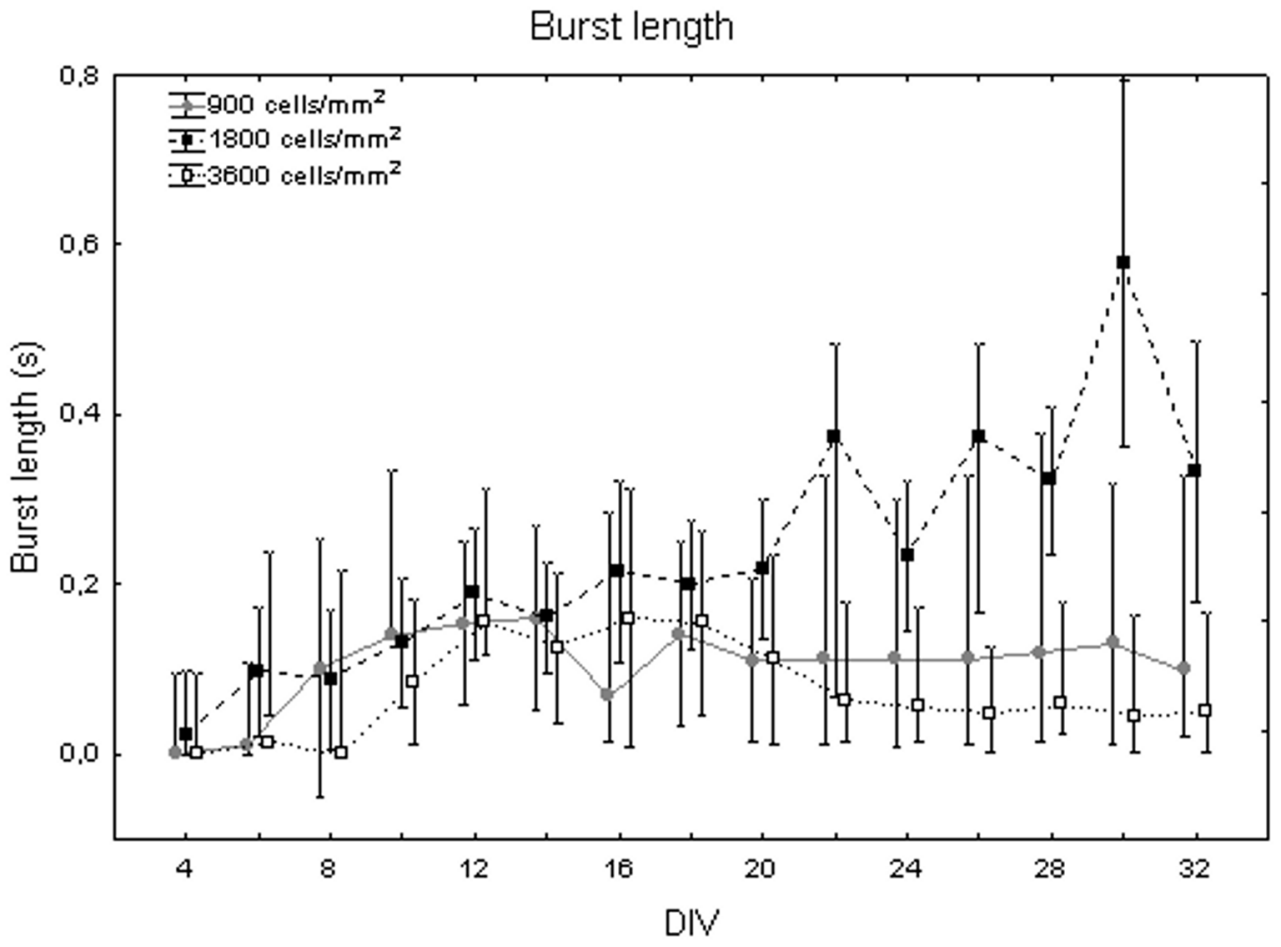
Changes of burst duration (s) during culture maturation at different cell densities. Neuronal culture density equal to 900 cells/mm^2^ (grey dots), 1800 cells/mm^2^ (black squares) and 3600 cells/mm^2^ (white squares).

Figure 8 shows the burst duration from 4 DIV to 32 DIV, considering median values for the cell densities. It displays an increasing trend during maturation, from 0+100 ms at DIV 4 to 160 ms +313 ms −15 ms at DIV 16 (Friedman test, p>0.05). Moreover, burst duration values vary with cell density (Kruskal-Wallis test, p<0.05): cell cultures at 3600 cells/mm^2^ (59 ms+60 ms −12 ms) significantly differ from cell cultures at 900 cells/mm^2^ (112 ms +22 ms −11 ms, Mann-Whitney U test p<0.05) and at 1800 cells/mm^2^ (215 ms +112 ms −68 ms, Mann-Whitney U test p<0.05) as shown in Figure 9. Looking at trends over time, burst duration increases during the first week *in vitro* for all the neuronal cultures remaining roughly constant for the rest of the time at densities of 900 and 3600 cells/mm^2^ (Figure 10, grey dots and white squares). By contrast, the burst length continuously increases at 1800 cells/mm^2^, reaching a duration equal to 600 ms (Figure 10, black squares) at day 30.

As described for the other parameters, the intra burst frequency smoothly increases with the maturation and remains stable after the end of the third week *in vitro* (Friedman test, p>0.05). On the whole, the median value of this descriptor at each density, grouping all the DIV together, is equal to 115 Hz +10 Hz−12 Hz with no differences between the three densities (Kruskal-Wallis test, p>0.05).

**Figure 11:**
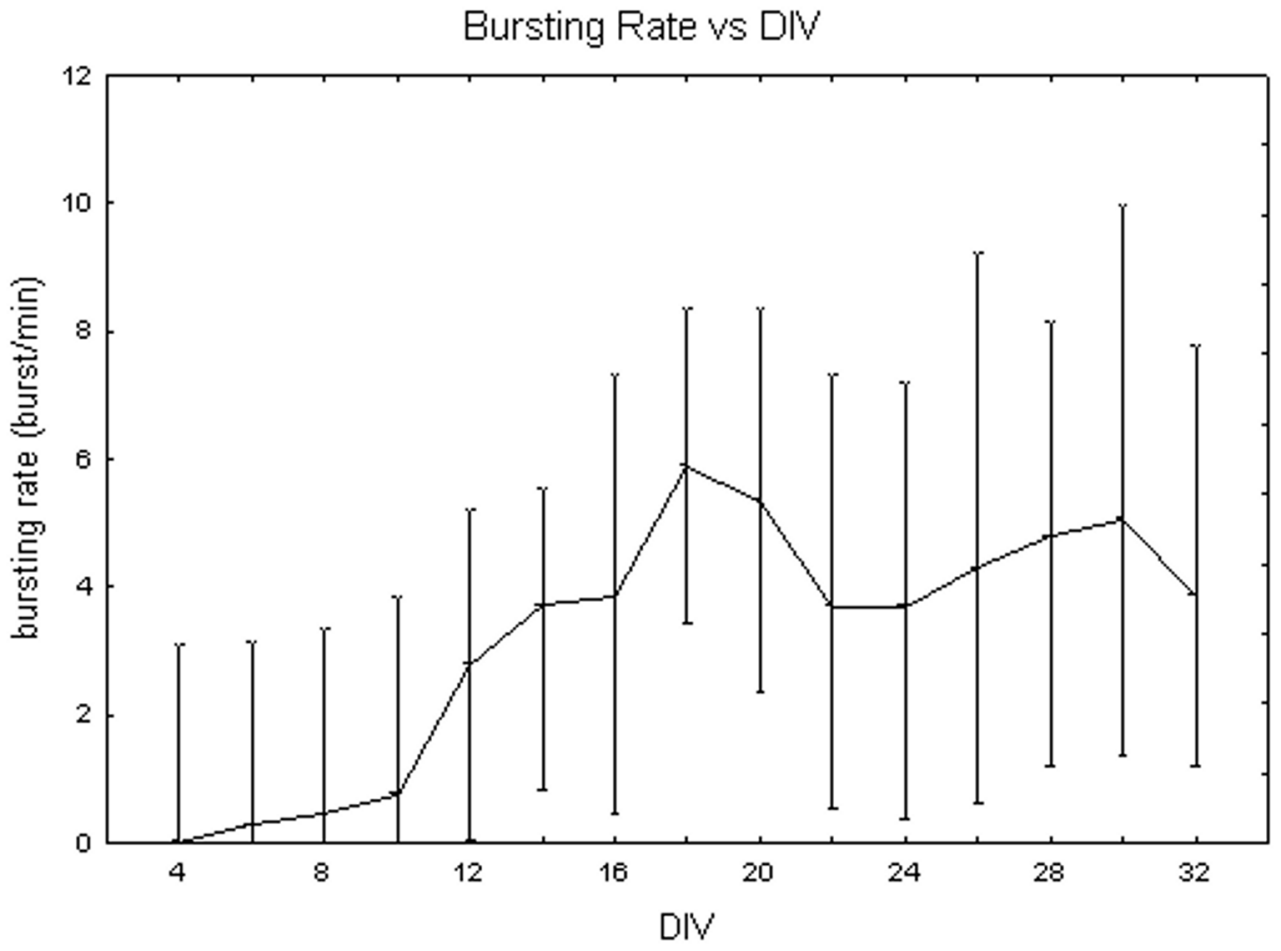
Changes of the bursting rate (bursts per minute) during cell culture maturation. Median values include different cell densities for each DIV.

**Figure 12:**
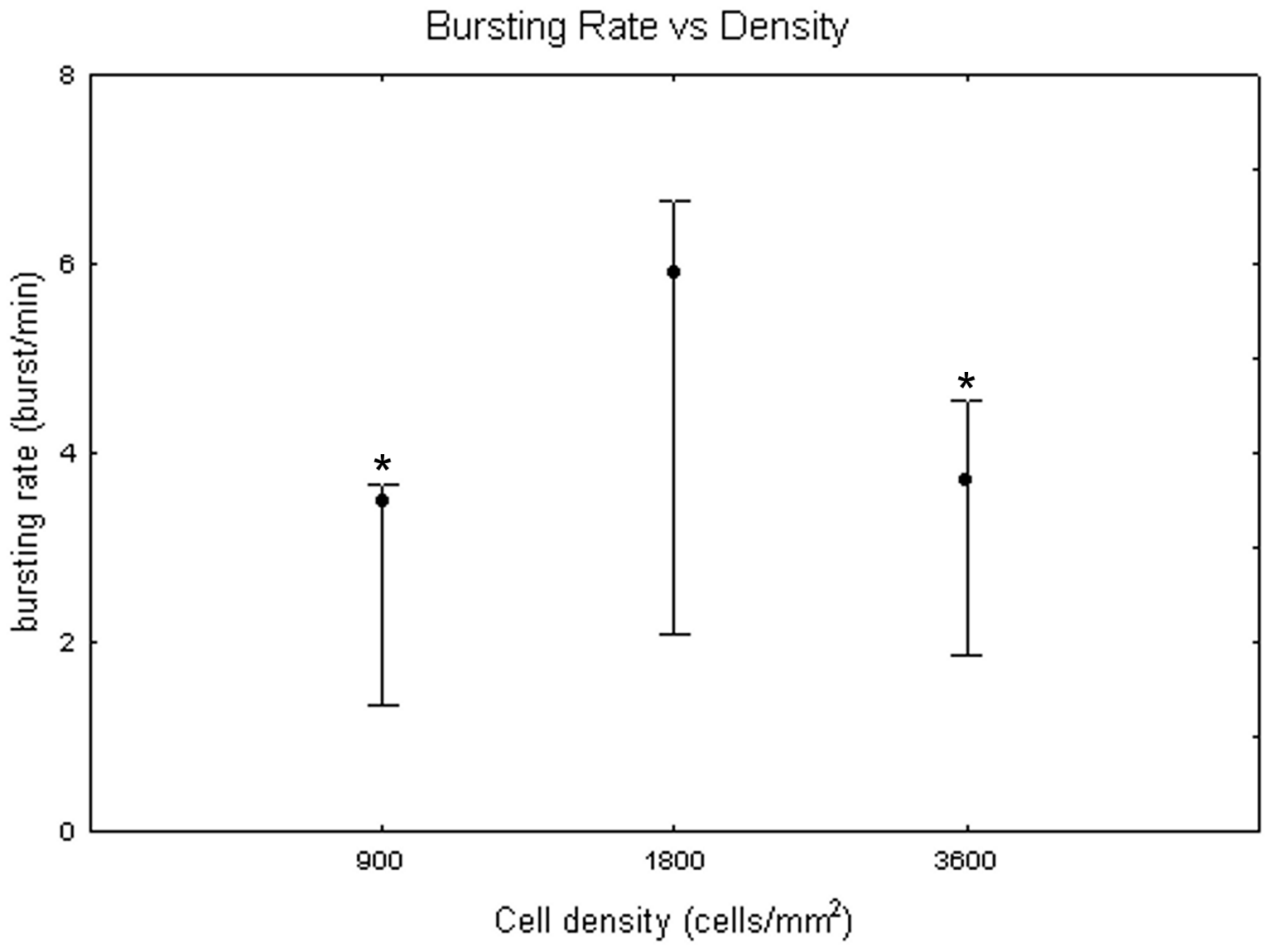
Bursting rate (bursts per minute) at 900, 1800 and 3600 cells/mm^2^. Median values include different DIV for each cell density. Kruskal-Wallis test and post-hoc Mann-Whitney U test *p<0.05 with respect to cell density of 1800 cells/mm^2^.

**Figure 13:**
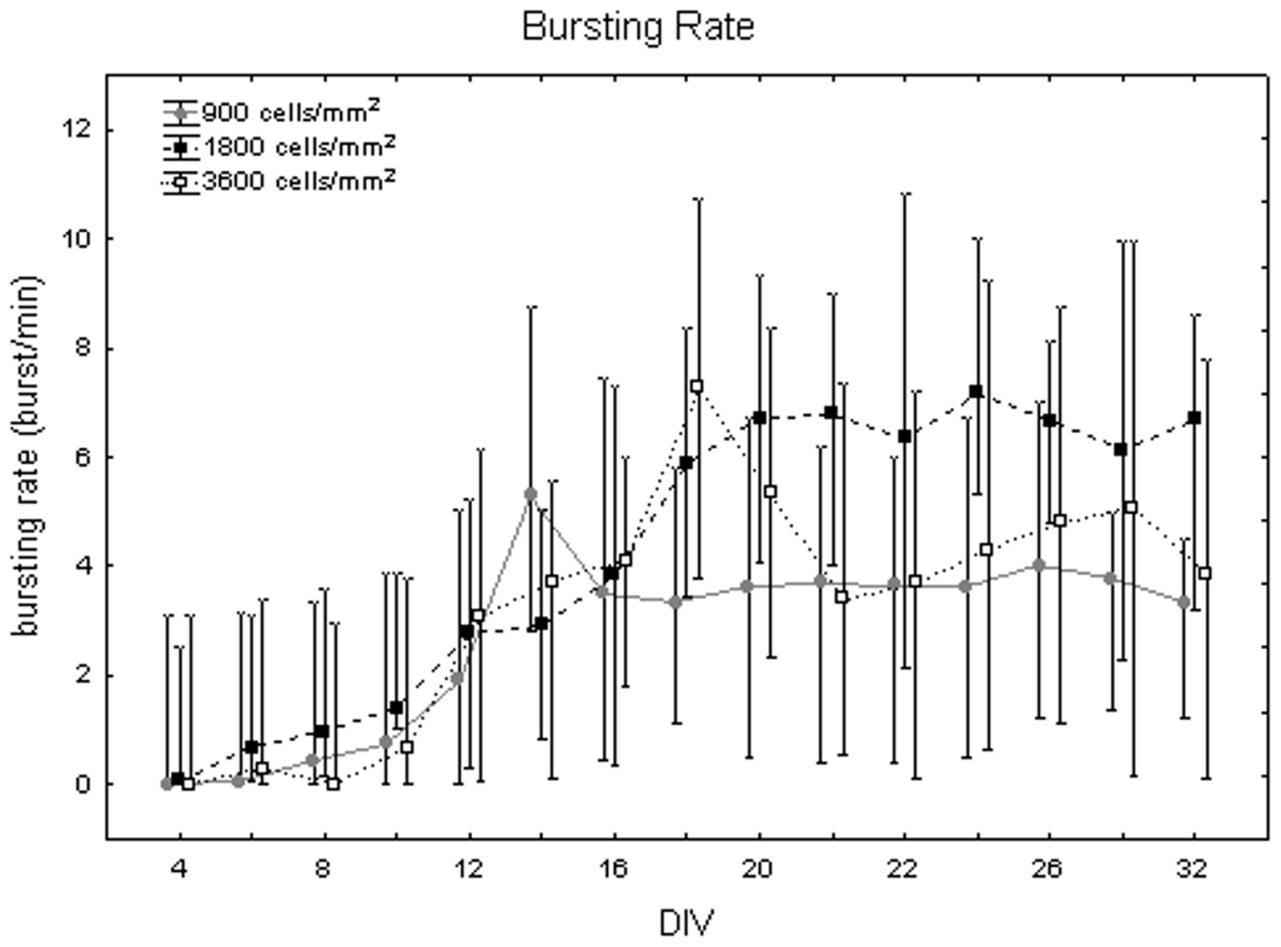
Bursting rate (bursts/min) during maturation at different cell densities. Cell culture density equal to 900 cells/mm^2^ (grey dots), 1800 cells/mm^2^ (black squares) and 3600 cells/mm^2^ (white squares).

Figure 11 represents bursting rate values at different DIV by showing the medians over all the densities. They vary between 0 to 5 bursts/min with a huge variability (even 7 bursts/min). No significant differences were identified between DIV with the Friedman test (p>0.05). In contrast, the Kruskal-Wallis test highlighted significant differences between the cell densities (p<0.05): the bursting rate is significantly higher for cell cultures at 1800 cells/mm^2^, reaching a value of 5.89 (+0.79 −3.80) bursts per minute, in contrast to the mean value of 3.5 (+0.18 −2.16) bursts per minute at 900 cells/mm^2^ (Mann-Whitney U test p<0.05) and of 3.71 (+0.84 −1.84) bursts per minute at 3600 cells/mm^2^ (Mann-Whitney U test p<0.05), as shown in Figure 12. Finally, Figure 13 shows the bursting rate considering both the DIV and the cell densities. From a qualitative point of view, the bursting rate increases during the first and the second week *in vitro* and it stabilizes for the rest of the experiment in neuronal cultures at 900 and 1800 cells/mm^2^ (Figure 13, grey dots and black squares, respectively). By contrast, cell cultures at 3600 cells/mm^2^ show a fast decrease of their bursting rate, after the peak at 18 DIV (Figure 13, white squares).

### Network Burst Features

**Figure 14:**
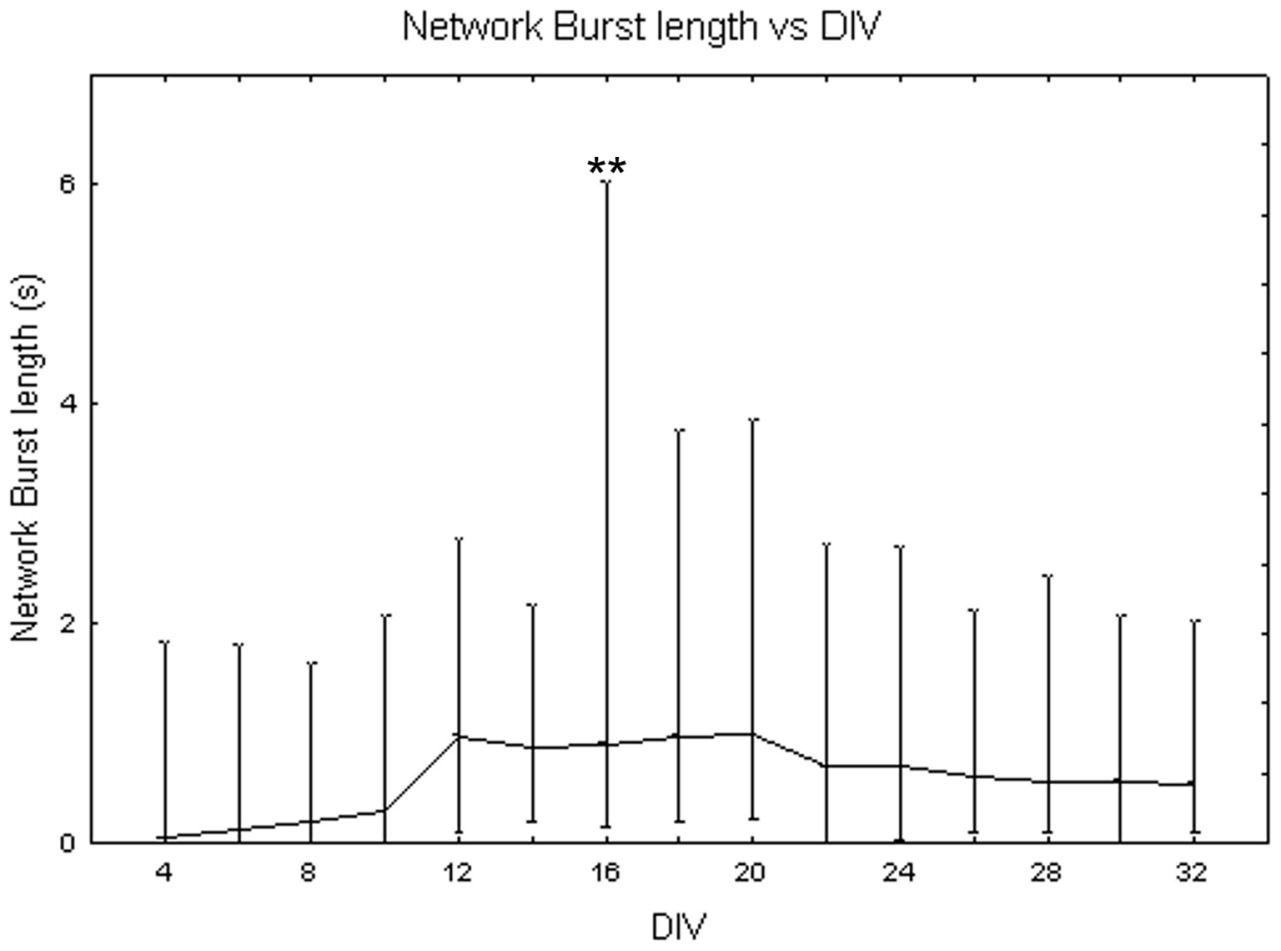
Changes of network burst length (s) during cell culture maturation. Median values include different cell densities for each DIV. One-way Friedman test and post-hoc Wilcoxon matched pair test **p<0.01 with respect to all the other days in culture.

**Figure 15:**
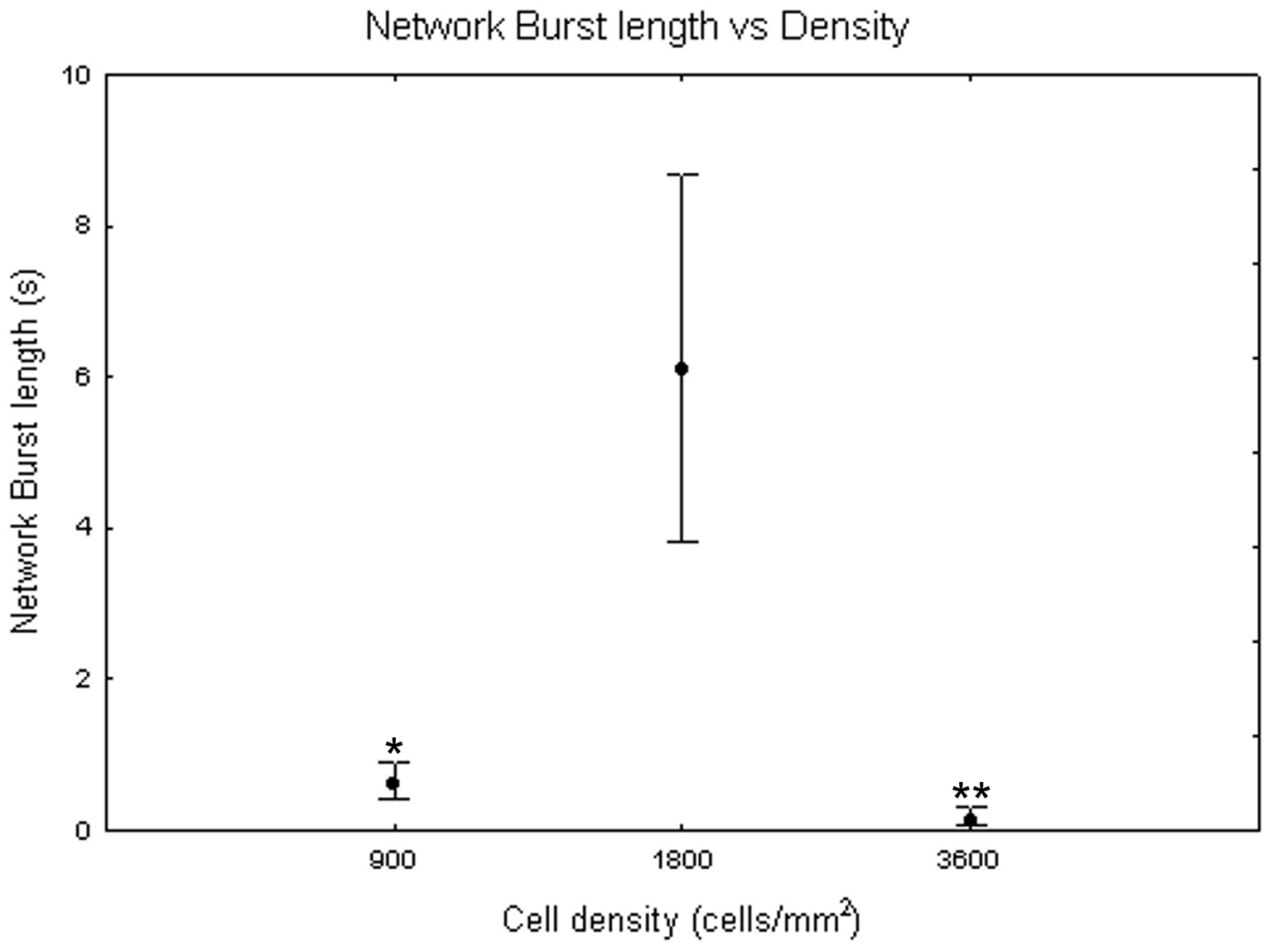
Network burst length (s) at 900, 1800 and 3600 cells/mm^2^. Median values include different DIV for each cell density. Kruskal-Wallis test and post-hoc Mann-Whitney U test *p<0.05 and **p<0.01 with respect to cell density of 1800 cells/mm^2^.

**Figure 16:**
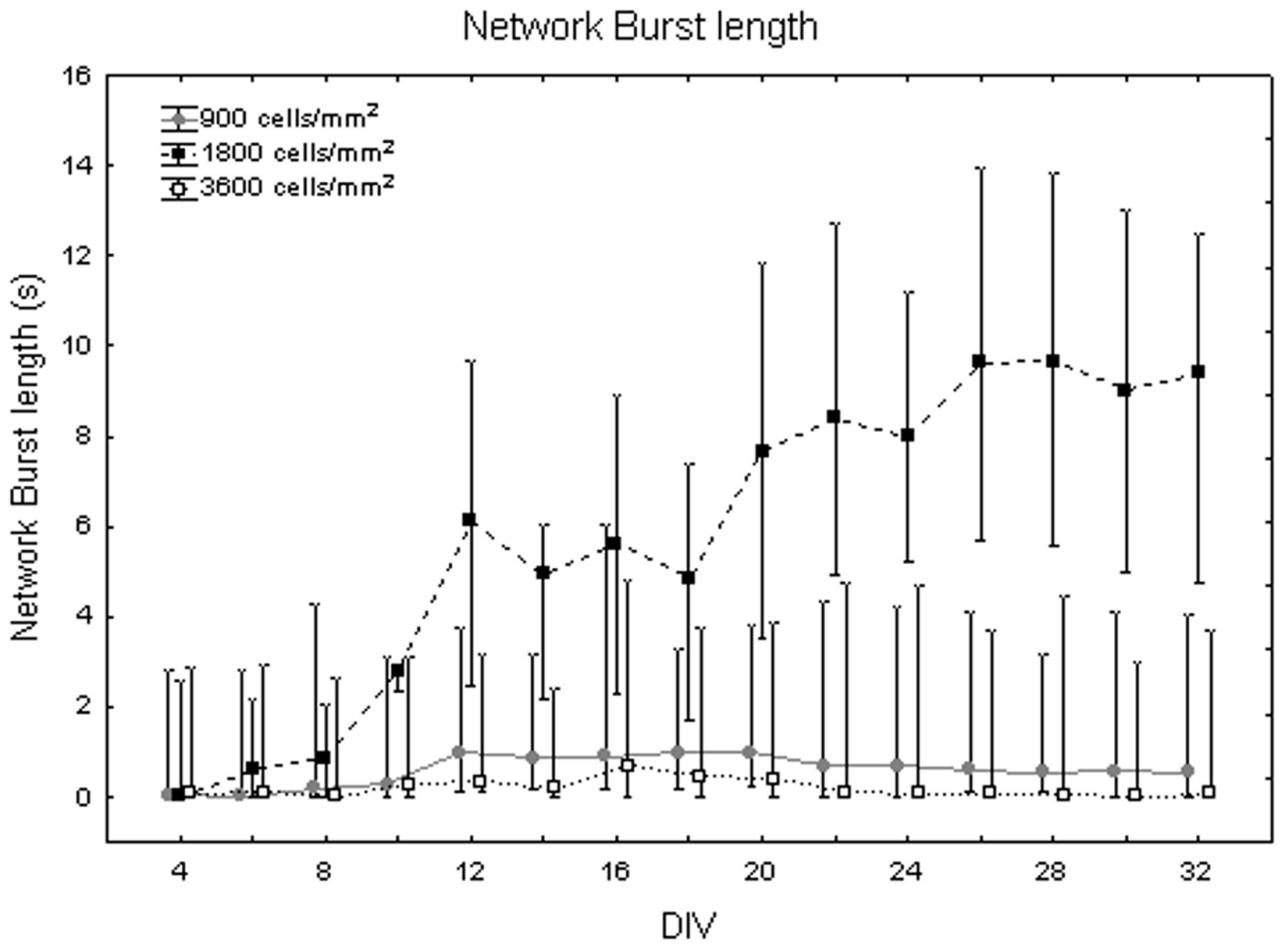
Changes of mean duration of NBs (s) during maturation at different cell densities. Neuronal culture density equal to 900 cells/mm^2^ (grey dots), 1800 cells/mm^2^ (black squares) and 3600 cells/mm^2^ (white squares).

Figure 14 illustrates the dependency of network burst (NB) length from the maturation. The one-way Friedman test showed significant differences between DIV (p<0.01) and the Wilcoxon matched pair test highlighted that these differences are between DIV 16 and all the other days of culture (p<0.01). Comparing cell densities, the Kruskal-Wallis test highlighted significant differences between them (p<0.05). Specifically, cell cultures at 1800 cells/mm^2^ (NB length = 6.11 s +2.60 s −2.30 s) differ from cell cultures at 900 cells/mm^2^ (NB length = 0.60 s +0.29 s −0.19 s, Mann-Whitney U test p<0.05) and at 3600 cells/mm^2^ (NB length = 0.11+0.21 −0.04 s, Mann-Whitney U test p<0.01) as shown in Figure 15. Analysing qualitatively the trend of network burst length for each cell density during maturation, it is possible to notice that it increases with neuronal network maturation, mostly for the density of 1800 cells/mm^2^ which reaches values of 10 s during the fifth week *in vitro* (Figure 16, black squares) and that is higher than neuronal networks at 900 cells/mm^2^ (Figure 16, grey dots) and 3600 cells/mm^2^ (Figure 16, white squares).

**Figure 17:**
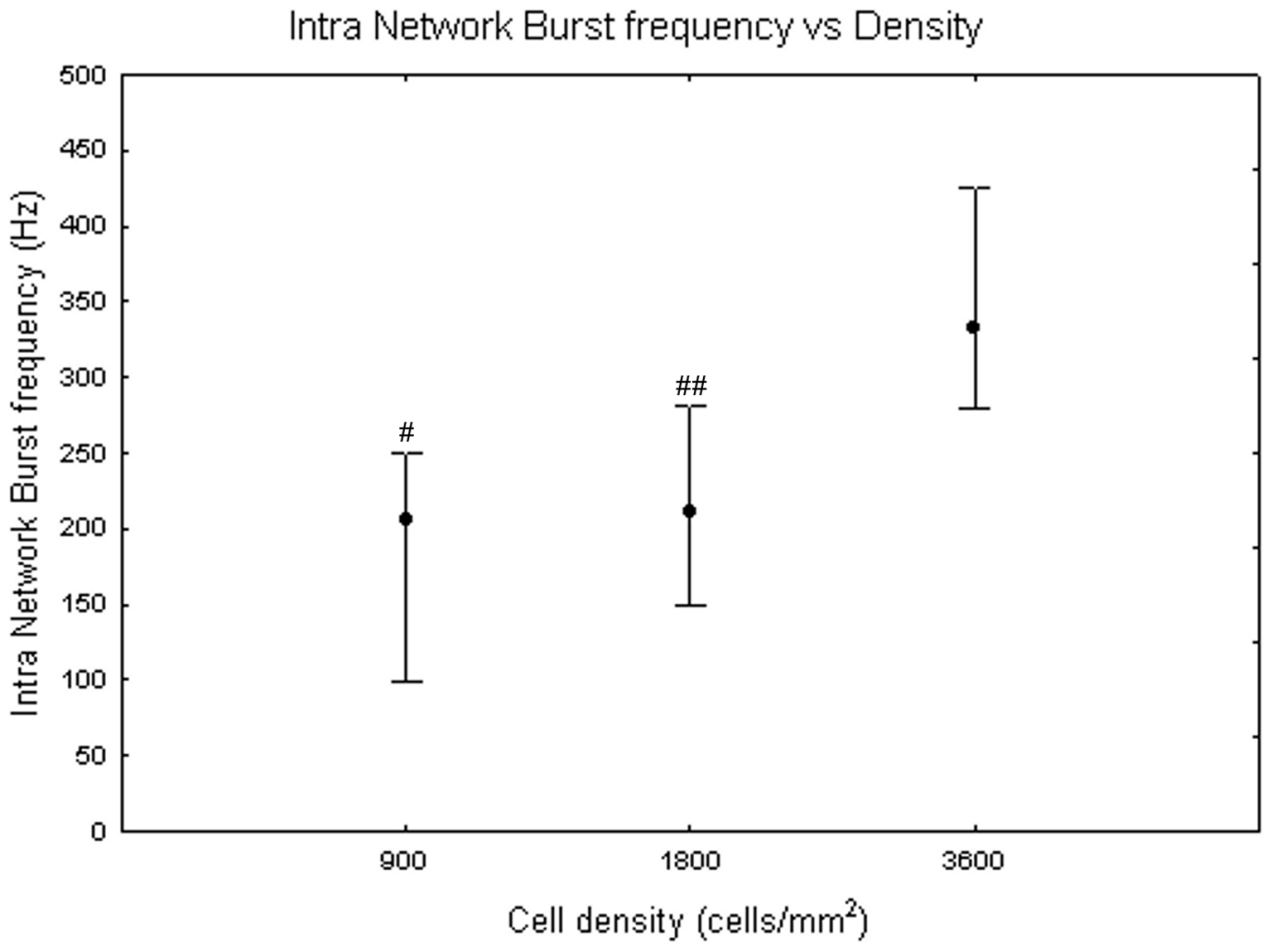
Intra network burst frequency at 900, 1800 and 3600 cells/mm^2^. Median values include different DIV for each cell density. Kruskal-Wallis test and post-hoc Mann-Whitney U test #p<0.05 and ##p<0.01 with respect to cell density of 3600 cells/mm^2^.

Concerning the intra network burst frequency, it has a trend similar to the network burst length during the maturation process, but without significant differences between DIV (Friedman test, p>0.05). By contrast, the Kruskal-Wallis test highlighted significant differences between cell densities (p<0.05). Specifically, cell cultures at 3600 cells/mm^2^ (333 Hz +91 Hz −52 Hz) differ from cell cultures at 900 cells/mm^2^ (205 Hz +44 Hz −106 Hz, Mann-Whitney U test p<0.05) and at 1800 cells/mm^2^ (211 Hz +71 Hz −61 Hz, Mann-Whitney U test p<0.01), as shown in Figure 17.

**Figure 18:**
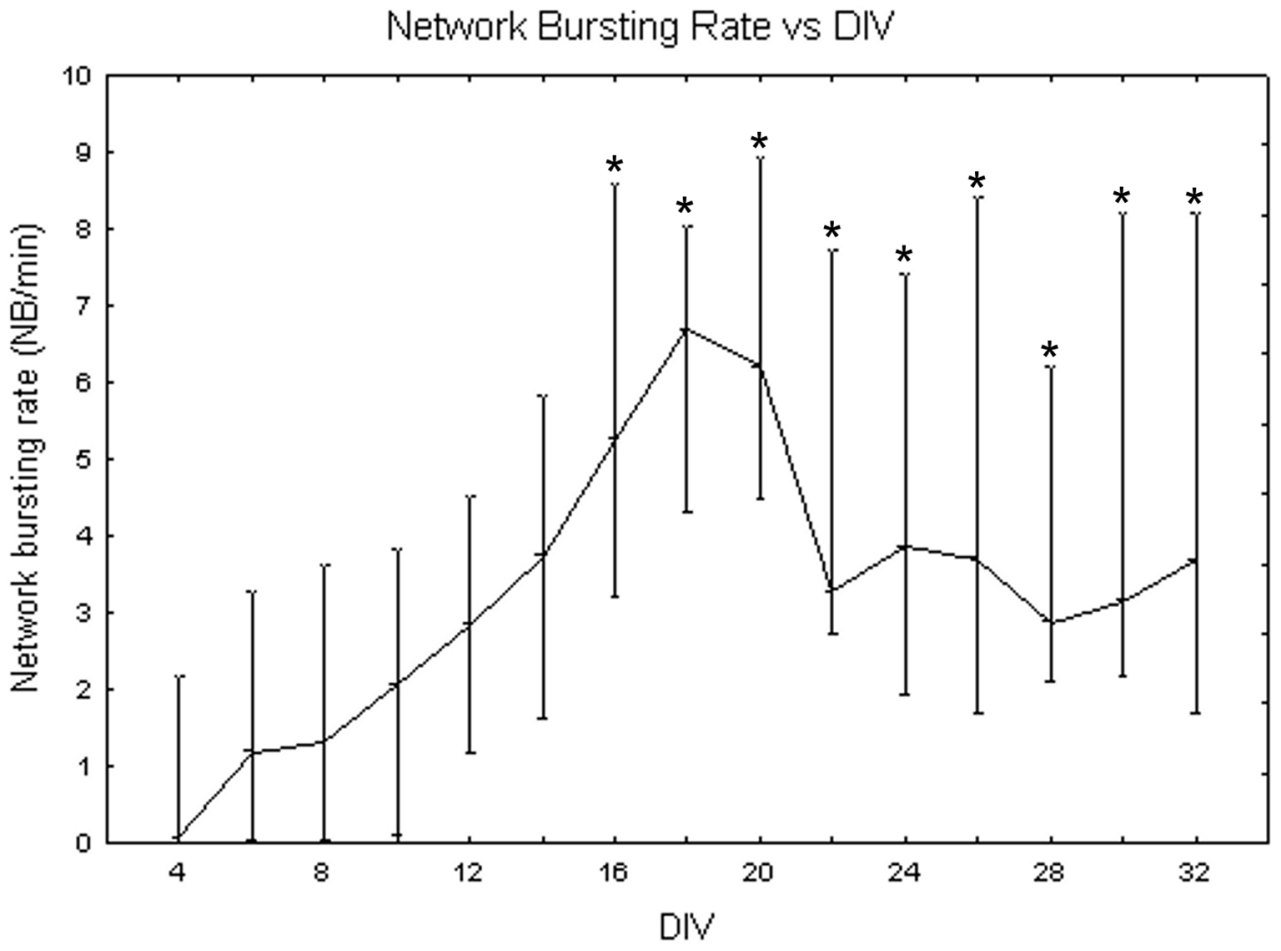
Network bursting rate (Hz) during cell culture maturation. Median values include different cell densities for each DIV. One-way Friedman test and post-hoc Wilcoxon matched pair test *p<0.05 with respect to the first and second weeks (from DIV 4 to DIV 14).

**Figure 19:**
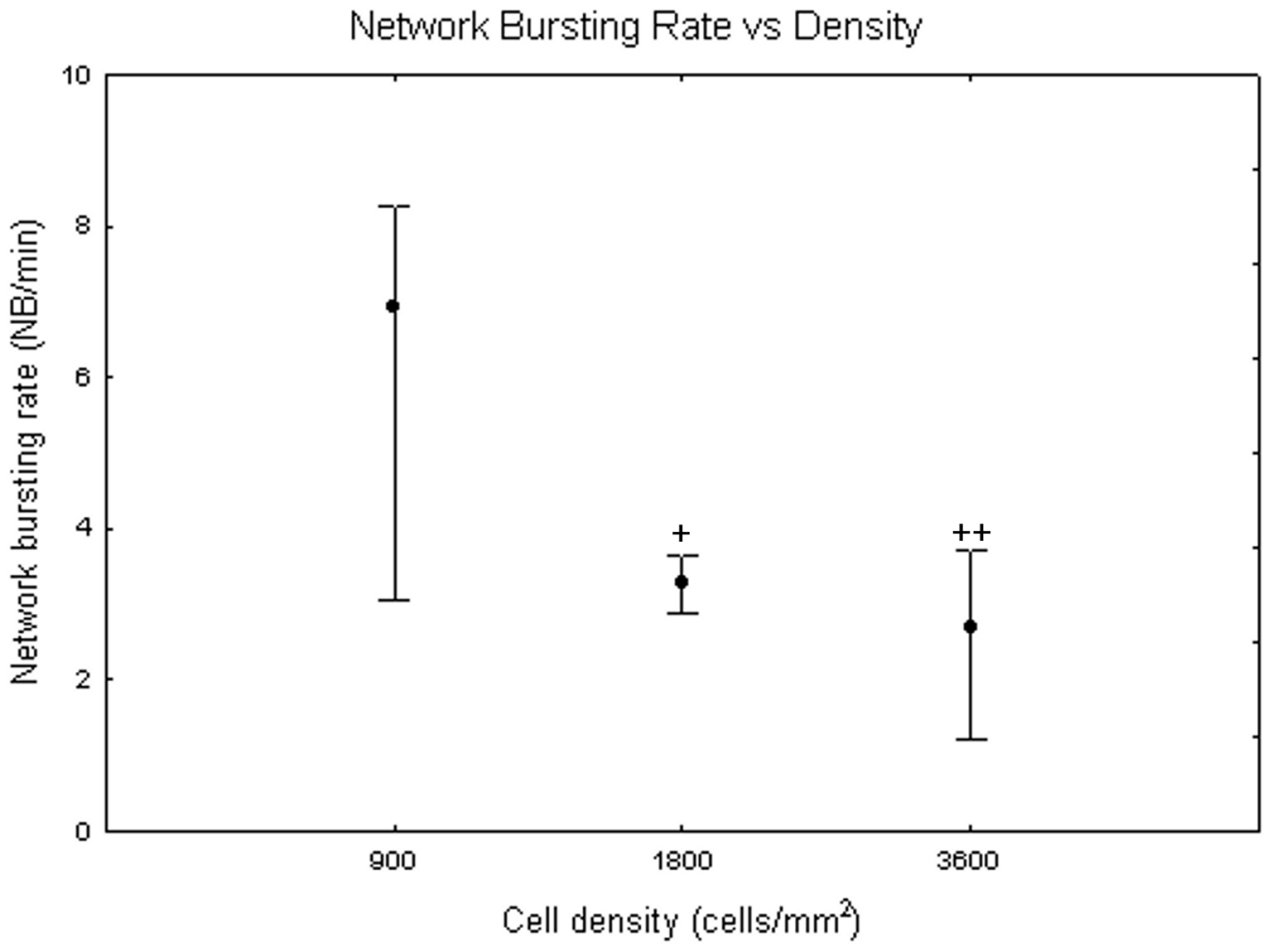
Network bursting rate (Hz) at 900, 1800 and 3600 cells/mm^2^. Median values include different DIV for each cell density. Kruskal-Wallis test and post-hoc Mann-Whitney U test +p<0.05 and ++p<0.01 with respect to cell density of 900 cells/mm^2^.

**Figure 20:**
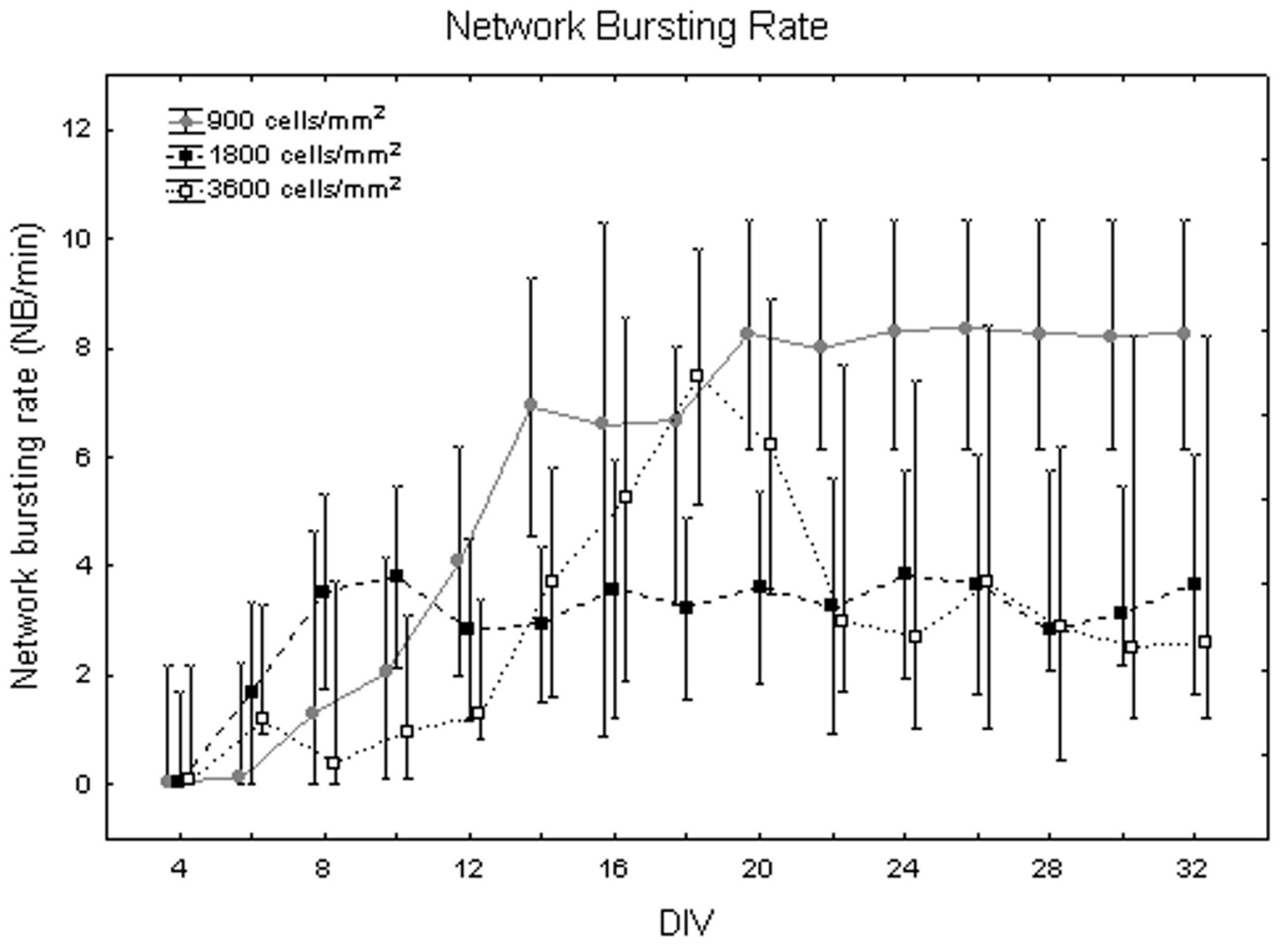
Network bursting rate (NB/min) during maturation at the three densities under study. Cell culture density equal to 900 cells/mm^2^ (grey dots), 1800 cells/mm^2^ (black squares) and 3600 cells/mm^2^ (white squares).

Figure 18 exemplifies the network bursting rate behaviour at different densities. The one-way Friedman test for repeated measures showed significant differences between DIV (p<0.05) and the Wilcoxon matched pair test highlighted that these differences are between the first and second weeks (from DIV 4 to DIV 14) and the third, fourth and fifth weeks (from DIV 16 to DIV 32, p<0.05). Concerning differences between densities, the Kruskal-Wallis test attested their presence (p<0.05). Specifically, cell cultures at 900 cells/mm^2^ (6.9+1.3 −3.9 NBs per minute) significantly differ from cell cultures at 1800 cells/mm^2^ (3.27+0.38 −0.37 NBs per minute, Mann-Whitney U test p<0.05) and at 3600 cells/mm^2^ (2.7+1 −1.47 NBs per minute, Mann-Whitney U test p<0.01) as shown in Figure 19. Qualitatively, neuronal networks at 900 cells/mm^2^, show a strong increase in the frequency of NB occurrences comparing the beginning of maturation to the fifth week *in vitro* (Figure 20, grey dots). By contrast, neuronal networks with a seeding density equal to 1800 cells/mm^2^ mature quite fast (during the first week) and then remain stable (Figure 20, black squares). Finally, at 3600 cells/mm^2^ the network bursting rate presents a peak around DIV 18 and then it decreases fast (Figure 20, white squares).

Finally, the percentage of spikes involved in NB, in contrast to free spikes, is similar for all the densities and it is equal to 27+15 −10%.

Table 2 summarizes the median values and percentiles for each cell density describing each electrophysiological descriptor analysed in the text. Median values include different DIV for each cell density.

**Table 2:** Median (M), 75^th^ percentile (P75) and 25^th^ percentile (P25) for each electrophysiological descriptor analysed and for each cell density (median values include different DIV for each cell density).

	900 cells/mm^2^			1800 cells/mm^2^			3600 cells/mm^2^		
	M	P75	P25	M	P75	P25	M	P75	P25
Nr active channels	0.33	0.4	0.28	0.55	0.62	0.5	0.39	0.48	0.33
Mean frequency (Hz)	0.89	1.73	0.72	2.13	2.74	1.92	0.67	1.5	0.15
Bursting channels (%)	35	40	25	44	47	30	21	25	13
Burst duration (ms)	112	134	101	215	327	147	59	119	47
Intra burst frequency (Hz)	115	125	103	115	125	103	115	125	103
Bursting rate (bursts/min)	3.5	3.68	1.34	5.89	6.68	2.09	3.71	4.55	1.87
NB duration (s)	0.6	0.89	0.41	6.11	8.71	3.81	0.11	0.32	0.07
Intra NB frequency (Hz)	205	249	99	211	282	150	333	424	281
NB rate (NBs/min)	6.9	8.2	3	3.27	3.65	2.9	2.7	3.7	1.23
Spikes in NB (%)	27	42	17	27	42	17	27	42	17

## Discussion

In this work we performed an experimental methodological study to assess the influence of neuronal density on the onset and evolution of the electrophysiological properties of maturing hippocampal cultures. The main purpose we intended to achieve is to provide reference data from mice hippocampal cultures grown on MEAs at different plating densities. We performed multisite measurements of the electrical activity of 86 independent neuronal networks plated at three different densities and we studied the influence of cell density on the electrical activity during maturation. Previous works have already characterized neuronal network maturation by means of extracellular recordings with MEAs [3], [6] but only at a single density or without providing clear indications about the functional characteristics of cultures plated with different densities to be used for experimental requirements. Due to the intrinsic variability of neuronal cultures *in vitro*, the indirect comparison of data at different densities, described in different works, is almost impossible, thereby suggesting the work here proposed. In this study, we have taken into account many descriptors, different densities and different DIV, giving a detailed, but simple, description of the evolution of spontaneous electrical activity of hippocampal neuronal networks with different culture densities. Culture-to-culture variation of the *in vitro* approach and the use of independent cultures assure that the reproducibility found in this work is a warranty of general reproducibility of the protocol.

As a starting summary of our results, the time profile of the electrophysiological descriptors retrieved from our cultures shows a similarity to the evolution of dissociated neuronal cultures found by previous works. Indeed, both the parameters describing the general level of activation of the network and the ones related to bursting and synchronized bursting behaviour increase until a peak around the end of the 2^nd^ or the beginning of the 3^rd^ week *in vitro*, which matches well with previous studies on hippocampal cultures [5], [11] and cortical cultures [3], [6]. Thus, our data confirm that the different origin of cultures does not imply a different spontaneous activity evolution during maturation.

Then, we chose to compare our results with two works taken from the literature, which better describe the effect of density on the electrophysiology of neuronal cultures. Our results concerning the influence of density on the electrical activity of hippocampal neuronal networks match with previous results on cortical cultures on MEAs by Wagenaar and colleagues. They clearly reported the electrophysiological behaviour of cultures at 600 cells/mm^2^ (sparse), 1600 cells/mm^2^ (medium) and 2500 cells/mm^2^ (dense), which are nearly comparable to our three densities, especially considering the great standard deviation that they obtained in the actual number of cells [6]. Indeed, we found that the trend of the activity descriptors is in agreement with that work, regarding the sparse and medium cultures (900 and 1800 cells/mm^2^). Specifically, we both found that sparse cultures display a lower firing rate over days than medium cultures and that medium cultures mature faster than sparse ones. This last information was obtained by observing that the age when the firing rate and the network bursts increased until a certain level (e.g., half of the peak) was higher for sparse cultures [6]. The same information can be easily retrieved by our data by looking at the time profile of the firing rate and network bursting rate for the sparse and medium cultures (Figure 7 and Figure 20). However, in contrast to that work, data related to the behaviour of the our dense cultures (3600 cells/mm^2^) show that they exhibit a weaker activity than the medium ones, and for some parameters similar to the sparse ones.

Cohen and co-workers analysed the activity of 2 week old hippocampal cultures seeded at three different densities (i.e., 380, 760 and 3800 cells/mm^2^) [11]. They found that the cell density of the network determines its properties, such that dense networks have higher rates of less synchronized activity than that of sparse networks, which are more synchronized but rhythm at lower rates. These results, obtained by means of calcium imaging, are in agreement with the trend of our MEA data at 900 and 1800 cells/mm^2^, which can be seen both in the data grouped by densities across DIV (Figure 11 and Figure 18) and in the time profile of busting rate (Figure 13) and network bursting rate (Figure 20) for the three densities after 14 DIV (median bursting rate equal to 4 and 7 bursts per minute; median network bursting rate equal to 8 and 4 NBs per minute at 900 and 1800 cells/mm^2^, respectively). Regarding the most dense cultures (3600 cells/mm^2^), in our testing conditions we found a discrepancy regarding the bursting rate parameter, which is significantly lower than in the medium dense cultures. Besides, the level of synchronization among different sites of the cultures has a lower median value, as found by [11], but not statistically relevant.

The differences about the most dense cultures with respect to [6] and [11] may be ascribed to some possible explanations. The first one is that cultures with very similar nominal densities may actually significantly differ from each other. Indeed, due to the tissue manipulation and culture conditions, the percentage of surviving cells after plating may be slightly different in the different laboratories, since the standard cell counting procedure (i.e., haemocytometer) is affected by high intra- and inter- experimenter variability. However, evidence from literatures suggest that the higher the nominal number of cells to count, the bigger the actual standard deviation of the number of cells may be [6] and so it can’t be excluded that the differences we noticed may be partly due to a different actual density. If this is true, the activity decline observed in the most dense cultures could be due to the fact that their density actually overcame an upper limit for long-term hippocampal cell viability in cultures. Indeed, the lower viability would explain the lower number of active sites and the lower firing and bursting rates. However, we observed that the few portions of the dense networks which continued emitting spikes across the culturing period exhibit a significantly higher intra network burst frequency, which can be explained by the fact that more cells near each electrode are recruited during network events compared to sparse and medium cultures. Even if the cell counting procedure used in this work (see Materials and Methods Section) is a suitable, robust reference for other experimenters, it may be refined in order to decrease the culture-to-culture variability in terms of cell number as well as the discrepancy between actual and nominal density. Moreover, experimenters should always be carefully trained to perform this procedure. A second factor which may likely have contributed to the decline observed in the dense cultures could be represented by the different culture feeding schedules used in our work. Indeed, we defined a feeding protocol and we applied it to all the cell density conditions. It is possible that a finer tuning of the fraction of medium to exchange is needed for high dense cultures to increase their longevity.

Moreover, it is worth highlighting that the work by Cohen and colleagues was performed by using a different experimental technique, which is calcium imaging. This technique has the advantage that it provides a finer spatial resolution compared to standard MEAs exploited in the present study and that it does not suffer from dishomogeneity of cells near the electrodes, but it does not allow repeated recordings from the same culture. The activity descriptors were averaged across all active neurons and not across all active electrodes. In this way, it is likely that when the culture is very dense the readout derives from a higher number of cells than with MEAs. This possible drawback linked to standard MEA technology should be considered in future studies.

It is also worth noting that all results in this manuscript are based on multiunit data. This choice was made since spike sorting algorithms are not still well established and reference techniques do not exist to this day. Moreover, spike sorting algorithms are complex and sometimes unreliable when many cells contribute to the spike train at each electrode. Indeed, we noticed that overlapping waveforms often occur during burst and network burst activity, making spike sorting problematic. Furthermore, since this work aims at giving a reference dataset, we do not want that the spike sorting procedure is required for further assessments. In this way, other researchers can easily compare their results with ours without the need of complex sorting algorithms.

What we have described here represents a reference work for the comparison of the spontaneous behaviour of neuronal networks at a certain cell density in terms of electrophysiological descriptors. The functional characteristics expressed at different densities can be useful for specific experimental purposes. Indeed, sparse cultures, which are characterized by a stable activity over the culturing period, seem to be adequate for long lasting experiments (e.g. chronic drug testing). Indeed, in chronic neuro-pharmacological applications it is important to have control cultures whose basal activity is stable throughout long periods. In this way, a researcher can test the effect of a drug treatment over many days by comparing it with a non-fluctuating reference signal. Moreover, the researcher does not need to get control cultures every experimental session, thus reducing the number of culture employed as control data. On the other hand, medium cultures seem to be more adequate for experiments that require intense electrical firing rates, as inhibitory treatments. Usually, in these kinds of experiments, the basal excitatory firing rate of the culture is partially suppressed by the drug under test. Therefore, an intense basal activity allows to appreciate even small modulation of electrophysiological features at high resolution. Finally, we observed that the activity of high density hippocampal cultures fluctuates and it is stable only around the second and the third week *in vitro*. Therefore, if researchers are planning to investigate high density network features, they should be aware of this limitation and they should accurately evaluate the timing of the experiment, preferring short time experiments, such as drug screening.
